# Climate-Based Models for Understanding and Forecasting Dengue Epidemics

**DOI:** 10.1371/journal.pntd.0001470

**Published:** 2012-02-14

**Authors:** Elodie Descloux, Morgan Mangeas, Christophe Eugène Menkes, Matthieu Lengaigne, Anne Leroy, Temaui Tehei, Laurent Guillaumot, Magali Teurlai, Ann-Claire Gourinat, Justus Benzler, Anne Pfannstiel, Jean-Paul Grangeon, Nicolas Degallier, Xavier De Lamballerie

**Affiliations:** 1 UMR190, Emergence of Viral Pathologies, Institute of Research for the Development, Aix-Marseille University, Marseille, France; 2 Department of Internal Medicine, Territorial Hospital Centre of New Caledonia, Noumea, New Caledonia; 3 UMR ESPACE-DEV 228, Institute of Research for the Development, Noumea, New Caledonia; 4 UMR 7159/UR 182, LOCEAN, Institute of Research for the Development, Noumea, New Caledonia; 5 UMR 7159/UR 182, LOCEAN, Institute of Research for the Development, University Paris VI, Paris, France; 6 Météo-France, Noumea, New Caledonia; 7 Laboratory of Medical Entomology, Pasteur Institute, Noumea, New Caledonia; 8 Laboratory of Virology, Pasteur Institute, Noumea, New Caledonia; 9 Public Health Surveillance and Communicable Disease Control Section, Public Health Division, Secretariat of the Pacific Community, Noumea, New Caledonia; 10 Health Department, Direction of Health and Social Affairs of New Caledonia, Noumea, New Caledonia; NASA Goddard Space Flight Center, United States of America

## Abstract

**Background:**

Dengue dynamics are driven by complex interactions between human-hosts, mosquito-vectors and viruses that are influenced by environmental and climatic factors. The objectives of this study were to analyze and model the relationships between climate, *Aedes aegypti* vectors and dengue outbreaks in Noumea (New Caledonia), and to provide an early warning system.

**Methodology/Principal Findings:**

Epidemiological and meteorological data were analyzed from 1971 to 2010 in Noumea. Entomological surveillance indices were available from March 2000 to December 2009. During epidemic years, the distribution of dengue cases was highly seasonal. The epidemic peak (March–April) lagged the warmest temperature by 1–2 months and was in phase with maximum precipitations, relative humidity and entomological indices. Significant inter-annual correlations were observed between the risk of outbreak and summertime temperature, precipitations or relative humidity but not ENSO. Climate-based multivariate non-linear models were developed to estimate the yearly risk of dengue outbreak in Noumea. The best explicative meteorological variables were the number of days with maximal temperature exceeding 32°C during January–February–March and the number of days with maximal relative humidity exceeding 95% during January. The best predictive variables were the maximal temperature in December and maximal relative humidity during October–November–December of the previous year. For a probability of dengue outbreak above 65% in leave-one-out cross validation, the explicative model predicted 94% of the epidemic years and 79% of the non epidemic years, and the predictive model 79% and 65%, respectively.

**Conclusions/Significance:**

The epidemic dynamics of dengue in Noumea were essentially driven by climate during the last forty years. Specific conditions based on maximal temperature and relative humidity thresholds were determinant in outbreaks occurrence. Their persistence was also crucial. An operational model that will enable health authorities to anticipate the outbreak risk was successfully developed. Similar models may be developed to improve dengue management in other countries.

## Author Summary

Dengue fever is a major public health problem in the tropics and subtropics. Since no vaccine exists, understanding and predicting outbreaks remain of crucial interest. Climate influences the mosquito-vector biology and the viral transmission cycle. Its impact on dengue dynamics is of growing interest. We analyzed the epidemiology of dengue in Noumea (New Caledonia) from 1971 to 2010 and its relationships with local and remote climate conditions using an original approach combining a comparison of epidemic and non epidemic years, bivariate and multivariate analyses. We found that the occurrence of outbreaks in Noumea was strongly influenced by climate during the last forty years. Efficient models were developed to estimate the yearly risk of outbreak as a function of two meteorological variables that were contemporaneous (explicative model) or prior (predictive model) to the outbreak onset. Local threshold values of maximal temperature and relative humidity were identified. Our results provide new insights to understand the link between climate and dengue outbreaks, and have a substantial impact on dengue management in New Caledonia since the health authorities have integrated these models into their decision making process and vector control policies. This raises the possibility to provide similar early warning systems in other countries.

## Introduction

Dengue viruses are the most important arthropod-borne viruses affecting humans. During the past century, the four serotypes (DENV 1 - DENV 4) have spread to about a hundred countries in the tropical and subtropical world including Asia, Africa, the Americas and the Pacific. Each year, an estimated 50 million people contract dengue fever with at least 500,000 cases of dengue haemorrhagic fever or dengue shock syndrome leading to 25,000 deaths [1]. The spatial distribution of this emerging infectious disease largely reflects the distribution of its primary urban mosquito vector, *Aedes aegypti* [2]. As no effective vaccine and specific treatment exist, vector control currently represents the only resource to mitigate dengue outbreaks.

Epidemic dynamics of dengue, like those of other vector-borne diseases, are driven by complex interactions between hosts, vectors and viruses that are influenced by environmental and climatic factors. Several determinants in dengue fever emergence have been identified including human population growth, accelerated urbanization, increased international transport, weakened public health infrastructure as well as a lack of effective vector control and disease surveillance [3]–[6]. On the other hand, there is growing interest in the impact of climate change on the emergence or re-emergence of vector-borne infectious diseases such as dengue [7]–[10]. It has been shown that climate-induced variations in modelled *A. aegypti* populations were strongly correlated to reported historical dengue cases (1958–1995) at the global scale [11], and a potential increase in the latitudinal and altitudinal distribution of *A. aegypti* and dengue are expected under global warming [5], [12].

In a specific ecosystem, the required conditions for the occurrence of a dengue outbreak include i) the presence of a dengue virus, ii) the presence and a sufficient density of competent vectors, iii) a sufficient number of susceptible humans that is serotype-specific, and iv) favorable environmental and climatic conditions for dengue transmission. Despite evidence that climate can influence dengue like other vector-borne diseases (i.e. vector population size and distribution, vector-pathogen-host interactions, and pathogen replication [7], ), the relationships between climate, *Aedes* mosquitoes density and behaviour, human populations and dengue incidence are not well understood.

Previous studies have shown that temperature influences the lengths of the mosquito gonotrophic cycle and the extrinsic incubation period of the virus within the mosquito, the survival rate of adults, the mosquitoes population size and feeding behaviours and the speed of virus replication [7], [13], [15]–[19]. Water is necessary for eggs and larva development, mosquito breeding, and humidity affects adult mortality [16]–[17], [20]–[22]. Temperatures and precipitations have been identified as influencing incidence rates of dengue in several endemic areas in the world (i.e. Thailand [23]–[24], Taiwan [25]–[27], Singapore [28], and Puerto Rico [24], [29]). On a broader scale, it is plausible that El Niño-Southern Oscillation (ENSO) also influences patterns of dengue transmission [23]–[24], [30]–[31]. This coupled ocean-atmosphere phenomena results in warm waters displacement and changes in sea surface temperatures (SST) across the Pacific Ocean, and has a strong influence on regional climates, particularly in the Pacific. ENSO can induce large temperature, humidity and precipitation changes for months (see the websites of the International Research Institute for Climate and Society (IRI, www.iri.org), and the National Oceanic and Atmospheric Administration (NOAA, www.noaa.gov) for more details). Importantly, previous studies revealed a positive correlation between ENSO, as measured by the Southern Oscillation Index (SOI), and dengue outbreaks in the South Pacific islands [30]–[31].

Our study was conducted in New Caledonia where dengue represents a major public health problem like in many Pacific Islands Countries and Territories [32]. The first dengue outbreak in New Caledonia occurred in 1884–1885 [33]. Disease transmission increased after World War II, and successive waves of epidemics involving all four serotypes were reported. Since 2000, serotype 1 has been predominant [34] causing more than 6,000 cases during the 2003–2004 epidemics [35] and about one thousand of cases in 2008. Although the serotype 4 [36] was involved in a major outbreak in 2009 (8,456 cases), the serotype 1 is still circulating. New Caledonia has had an effective surveillance system for dengue and access to high quality meteorological data for many years. Since 2000, regular entomological surveillance is performed. This provides an opportunity to study the influence of climate variations on dengue dynamics.

We analyzed the epidemiology of dengue fever in Noumea, the capital of New Caledonia, from 1971 to 2010 together with local and remote climate influences. The objectives of this study were i) to improve our knowledge of the relationships between meteorological variables, entomological surveillance indices and dengue fever dynamics at seasonal to inter-annual time scales, ii) to identify suitable conditions for an epidemic occurrence, and iii) to develop a predictive model for dengue outbreaks that can be integrated in an early warning system in New Caledonia.

## Methods

### Study area

New Caledonia is a French overseas territory located in the subregion of Melanesia in the southwest Pacific, about 1,200 kilometres east of Australia and 1,500 kilometres northwest of New Zealand. It lies astride the Tropic of Capricorn, between 19° and 23° south latitude. Its climate is tropical.

This archipelago of 18,575 square kilometres is made up of a main mountainous island elongated northwest-southeast 400 kilometres in length and 50–70 kilometres wide, the Loyalty Islands (Mare, Lifou, and Ouvea), and several smaller islands (e.g. Isle of Pines). The population was estimated in January 2009 to be 245,580 [37]. Approximately half of inhabitants are concentrated in the southeast region of the main island around Noumea, the capital.

*A. aegypti* is the only mosquito vector of dengue in New Caledonia. The two others vectors of dengue present in the Pacific region, *A. albopictus* and *A. polynesiensis*, have never been detected in this archipelago [38]–[40]. In Noumea, most of *A. aegypti* breeding sites are outdoors and therefore rainfall dependent.

### Data collection

#### Epidemiological data

All cases of dengue fever and dengue haemorrhagic fever reported from January 1971 to December 2010 were collected from the Pasteur Institute, the Health Department of the Direction of Health and Social Affairs of New Caledonia, and the Communicable Disease Surveillance Division, Secretariat of the Pacific Community. A clinical case was defined as sustained fever and at least two of the following criteria: nausea or vomiting, myalgia or arthralgia, headache or retro-orbital pain, rash and/or spontaneous bleeding. A laboratory positive case was defined as a single or paired serum sample positive in serological assays (hemagglutination inhibition, IgM detection by indirect immunofluorescence or ELISA), or direct detection of dengue virus by reverse-transcriptase polymerase chain reaction (RT-PCR using a pandengue technique), virus isolation, or NS1 antigen detection (ELISA or rapid immunochromatographic test). The serotyping of positive samples using RT-PCR with specific primers for DENV-1, 2, 3, and 4 was regularly performed at the Pasteur Institute over the 1971–2010 period.

Since 1995, a georeferencing of dengue cases per council has been performed. To identify the possible origin of dengue infection, travel history and first day of illness were determined by the Health Department of the Direction of Health and Social Affairs of New Caledonia. Imported dengue cases were defined as laboratory positive dengue cases with travel history to endemic countries within 14 days before the date of disease onset.

**Figure 1:**
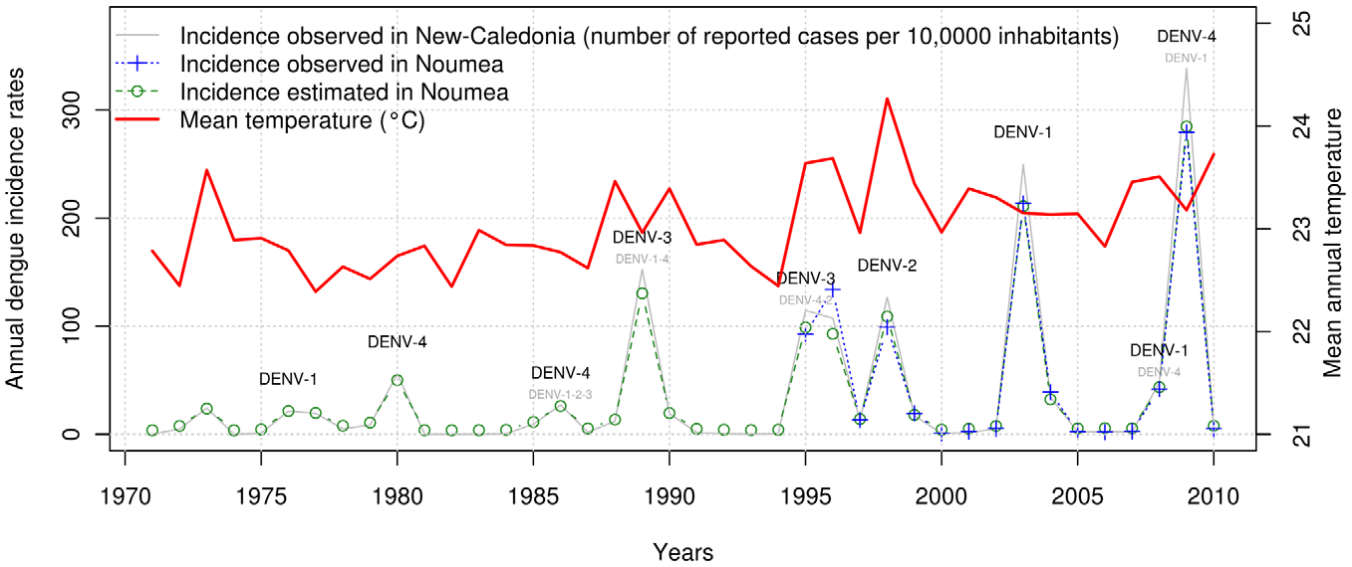
Epidemiology of dengue fever and evolution of annual mean temperature in Noumea-New Caledonia (1971–2010). The predominant circulating serotype (DENV-1, DENV-2, DENV-3 or DENV-4) is indicated in black characters. When other serotypes were detected, they are indicated in little grey characters. Annual dengue incidence rates observed in Noumea over the 1995–2010 period are highly correlated with dengue incidence rates observed in New Caledonia (Spearman coefficient *rho* = 0.99, *p*-value = 1*10^−14^). Annual dengue incidence rates in Noumea (1971–1994) were estimated (green dotted line with circles) on the basis of the relationship between incidence rates observed in New Caledonia (grey line) and those observed in Noumea (blue dotted line with crosses) using a linear model. During the 1971–2010 period, dengue incidence rates and annual mean temperatures (from January to December) were significantly correlated in Noumea (Spearman's coefficient *rho* = 0.426, *p*-value = 0.007). An increasing trend of dengue outbreaks amplitude and annual mean temperatures were observed during this 40-year study period.

Incidence rates of dengue were calculated each year in New Caledonia (number of dengue cases per 10,000 inhabitants per year) using population data based on linear extrapolations of local census reports (1969, 1976, 1983, 1989, 1996, 2004, 2009 [37]). In Noumea, annual dengue incidence rates were computed for the 1995–2010 period from observed data. As georeferencing was not available before 1995, and as there is a strong linear relationship between incidence rates observed in Noumea and those observed in the entire territory over the 1995–2010 period, we used a linear fit to estimate dengue incidence rates in Noumea for the 1971–1994 period (Figure 1).

Two methods were used to separate the years during which dengue outbreaks occurred (denoted epidemic years) or did not occur (denoted non epidemic years) on the basis of annual incidence rates of dengue cases computed from January to December in Noumea. The first method denoted “tercile method” divided the years into three groups: epidemic years when the dengue incidence rate belonged to the upper tercile, non epidemic years when the dengue incidence rate belonged to the lower tercile, and unclassifiable years when the dengue incidence rate belonged to the central tercile. The second method denoted “median method”, divided years into two groups: epidemic years when the annual incidence rate was greater than the median of the annual dengue incidence rates over the 1971–2010 period, and non epidemic years when the annual incidence rate was lower than the median. The first method allowed the problem of epidemic threshold to be minimised and to ensure a clear separation between epidemic and non epidemic years but with a 30% data loss while the second one allowed models to be built using the whole set of data.

#### Meteorological data

Two types of meteorological data were used: meteorological data measured at the reference weather station of Météo-France in central Noumea, and ENSO indices.

Data collected at the Noumea weather station for the period January 1971 to December 2010, the time period of the available dengue data, were analyzed. This station provides observations that are representative of the local climate around Noumea which contributes the most dengue cases in New Caledonia (Figure 1), and where dengue outbreaks usually begin. From these daily data, monthly, quarterly and annual means were calculated as well as monthly and quarterly number of days with a daily parameter greater than a given threshold. Quarterly data were generated with a sliding window each month. Monthly and quarterly parameters were named “parameter_month”, and “parameter_first letter of each month of the quarter”, respectively. The meteorological parameters of interest were daily minimum, mean, and maximum temperatures (min Temp, mean Temp, max Temp), daily minimum, mean, and maximum relative humidity (min RH, mean RH, max RH), and cumulative precipitations (Precip). Other parameters that may influence the productivity of larval breeding sites and mosquitoes populations were also considered such as mean daily wind force at 10 meters (WF), potential evapotranspiration by Penman-Monteith (ETP) and potential hydric balance sheets (HB = Precip-ETP) reflecting water resources. Numbers of days with a parameter over a threshold *x* were named NOD_parameter_threshold *x*. Several temperature thresholds were analyzed for min Temp, mean Temp and max Temp, ranging from 21 to 25°C, 24 to 28°C, and 25 to 35°C, respectively. Different thresholds were also analyzed for min RH (50%, 60%, 70%), mean RH (70%, 80%) and max RH (80%, 90%, 95%), for Precip (0.1, 1, 2, 3, 4, 5, 10, 25 mm/day), ETP (4, 5, 6, 7), HB (−5, 0, 5, 10 mm), and WF (3, 4, 5, 6, 7, 8 m/s).

Several ENSO indices were integrated in the analysis: Niño 3, Niño 3.4, Niño 4, Southern Oscillation Index (SOI), and Multivariate ENSO Index (MEI). Time series of these monthly ENSO indices were obtained from the NOAA Climate Prediction Center [41].

Altogether approximately 4000 meteorological data were generated for the 1971–2010 period (monthly, quarterly, and annual values). They were aggregated in seven families: temperature, relative humidity, precipitations, wind force, potential evapotranspiration, hydric balance sheet and ENSO.

#### Entomological surveillance data

An entomological surveillance network was established since 1997 at the initiative of the Pasteur Institute, the Health Department of the Direction of Health and Social Affairs of New Caledonia, and councils of Noumea and its neighbouring towns.

Since March 2000, about one hundred randomly selected houses in each of three districts of Noumea (East, West, and South) in a homogeneous and representative panel of 6,608 houses were visited each month to determine the number and type of larval developmental places, and the number of *A. aegypti* larvae, pupae and female adults. The following surveillance indices were computed monthly by the Laboratory of Medical Entomology of the Pasteur Institute:

- House Index (HI) = number of houses with at least one larval breeding site positive for *A. aegypti* x 100)/number of inspected premises.
- Breteau Index (BI) = number of larval breeding sites positive for *A. aegypti*/100 inspected premises.
- Adult Productivity Index (API) = number of *A. aegypti* pupae and stage 4 larvae/number of inspected premises.

### Statistical analysis and modelling

Bivariate and multivariate analyses were conducted using the R software package (R development Core Team version 2.9.1 [42]).

#### Time series analysis

Time series analysis of monthly, quarterly and annual data of dengue incidence rates, entomological indices and climatic variables were studied. Their temporal evolution was studied at inter-annual and seasonal scales. Global trends were computed for epidemiological and meteorological time series using linear regression (trend line).

#### Bivariate analysis

The relationships between epidemiological and meteorological data, entomological and meteorological data, and entomological and epidemiological data were studied in Noumea at different time-scales using a Spearman's method with *p*-values below 0.05 indicating statistical significance. At the annual scale, time series of annual dengue incidence rates and annual means of meteorological variables were analyzed from 1971 to 2010. At the monthly scale, time-lagged correlation analyses (lag being equal to 0, 1, 2 and 3 months) were performed on time series of monthly means of meteorological variables, entomological indices and dengue incidence rates from March 2000 to December 2009.

#### Comparative analysis of epidemic years and non epidemic years

To minimize the influence of changes in disease surveillance and diagnosis over the 1971–2010 period, we decided to use series of epidemic years (0 for non epidemic years, 1 for epidemic years, according to the tercile method described above) rather than dengue incidence rates.

Epidemic and non epidemic years were compared to identify suitable seasonal meteorological patterns for dengue outbreak occurrence. Monthly and quarterly meteorological data observed in Noumea during epidemic and non epidemic years were compared from August (year *y*-1) to July (year *y*) and means and 95% confidence interval (IC95%) were calculated. Categorical variables were compared using a two-sided *t*-test and correlation analyses were performed using a Spearman's rank correlation test. The *p*-values below 0.05 were considered to indicate statistical significance.

#### Multivariate modelling of dengue outbreak risk

The final objective of this study was to design two types of model to predict the risk of dengue outbreak in Noumea. The first model named hereafter “explicative model” was expected to identify suitable conditions for an epidemic occurrence using data from September (year *y*-1) to April (year *y*), i.e. four months before and after the outbreak onset (in January). The second model named hereafter “predictive model” was intended to help the health authorities of New Caledonia to anticipate the risk of a dengue outbreak. Only meteorological variables available prior to the outbreak onset, i.e. from September (year *y*-1) to December (year *y*-1) were used in this framework. On the basis of the bivariate analysis results, we decided to focus on the monthly and quarterly meteorological data. Poorly correlated variables such as wind force were excluded from the pool of potential input variables.

The type of classification method used for both explicative and predictive models was the Support Vector Machines (SVM) which is a supervised pattern recognition technique recently introduced in Statistical Learning Theory [43]. The main advantage of this method is that SVM are based on the principle of Structural Risk Minimization rather than on the error rates as do many other methods. SVM focus on generalizing well rather than correctly classifying the training dataset (i.e. minimizing the generalization error rather than the training error). The concept of SVM is to design a function which correctly classifies all of the objects of the training dataset. In the linearly separable case, SVM allow the identification of an hyperplane which is defined by the following equation: ***w*** *.x*+*b* = 0 where ***w*** is a vector normal to the hyperplane and *b* is the bias. In the non linear case, the separating surface is found by mapping the input points onto a higher dimensional space where the training dataset become linearly separable and by using an appropriate kernel (here a Gaussian kernel) in the optimization process [43].

In our study, the SVM took as input a set of meteorological data and predicted, for each given input, which one of the two possible classes the input is a member (epidemic year or non epidemic year). All the available data (40 years) were used for training the model and the median method, introduced above, was applied to separate the years. The results were then supplied as probability estimates of dengue outbreak occurrence using the method developed by Wu et al. [44].

The selection of the most relevant model was achieved using a forward stepwise selection method based on the corrected Akaike Information Criterion (AIC_c_) [45]–[46]. This method not only rewards goodness of fit, but also includes a penalty that discourages overfitting.

The robustness of the explicative and predictive models was estimated using a leave-one-out cross validation method: a single observation (year *y*) from the original sample (1971–2010 years) was retained as a validation data for testing the model, and the remaining observations were used as training data. This process was repeated 40 times such that each yearly observation in the sample was used once as the validation data. The results from the folds then were averaged to produce a single estimation of dengue outbreak risk in Noumea each year. The performance of the models was estimated with the Receiver Operator Characteristics - Area Under the Curve (ROC-AUC). The sensitivity, specificity, positive predictive value and negative predictive value were calculated for each model.

## Results

### Time series analysis

#### Dengue data

During the 1971–2010 period, successive waves of dengue outbreaks involving the four serotypes were recorded in New Caledonia with an increasing magnitude, particularly in Noumea where dengue outbreaks usually begin (Figure 1). The annual dengue incidence rates revealed a global upward linear trend (mean increase of 65.4 dengue cases per 10,000 inhabitants over the studied period in Noumea). The most severe outbreaks were caused by DENV-1 and more recently DENV-4 in 2003 (5673 reported cases, 733 hospitalizations, 19 deaths), 2008 (1170 reported cases, ∼100 hospitalizations, two deaths) and 2009 (8456 reported cases, 470 hospitalizations, three deaths). On four occasions, dengue outbreaks were repeated in two successive years: in 1976–1977 (DENV-1), 1995–1996 (DENV-3), 2003–2004 (DENV-1), and 2008–2009 (DENV-1 and DENV-4).

**Figure 2:**
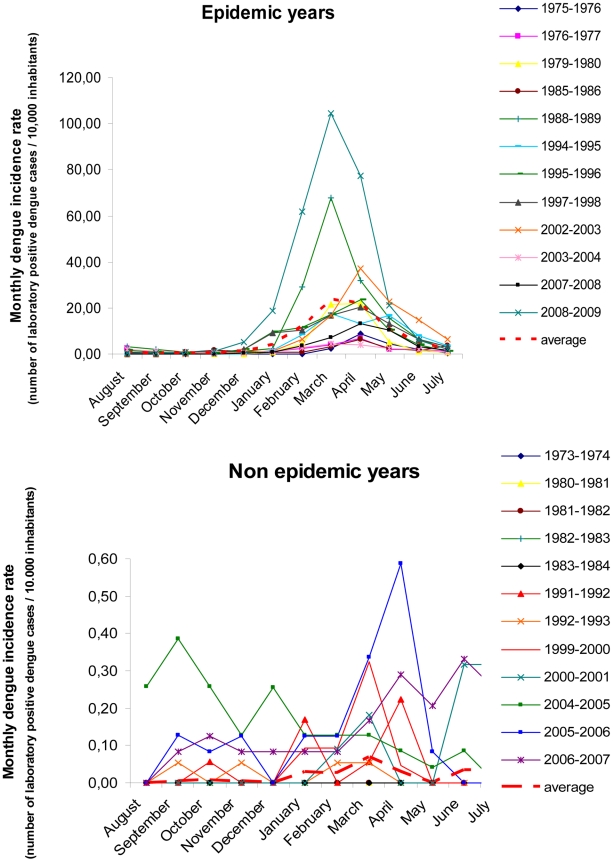
Monthly distribution of laboratory positive dengue cases during epidemic and non epidemic years. A strong seasonality in the dengue cases distribution was observed during epidemic years with outbreaks occurring usually between January and July. By contrast, dengue cases occurred almost every month without a clear seasonal pattern during non epidemic years.

The analysis of monthly reported and laboratory positive cases revealed a strong seasonal distribution of dengue cases during epidemic years (Figure 2). The majority of outbreaks displayed a similar seasonal evolution: beginning in January, an epidemic peak between March and May, and ending in July. The temporal distribution of dengue cases during non epidemic years was different, with an occurrence of cases every month. Imported dengue cases from different locations in Asia and the Pacific (particularly Indonesia, the Philippines and French Polynesia) were recorded once or several times a year without a clear seasonal pattern.

#### Entomological data

Entomological surveillance data were available from March 2000 to December 2009 in Noumea and a decreasing trend of all entomological indices was observed (supporting Figure S1). Indices reflecting the distribution and the abundance of larval developmental places (HI and BI), and the vector density (API) were strongly correlated (HI versus BI: *rho* = 0.98, *p*-value<0.001; API versus HI: *rho* = 0.82, *p*-value<0.001; API versus BI: *rho* = 0.84, *p*-value<0.001).

**Figure 3:**
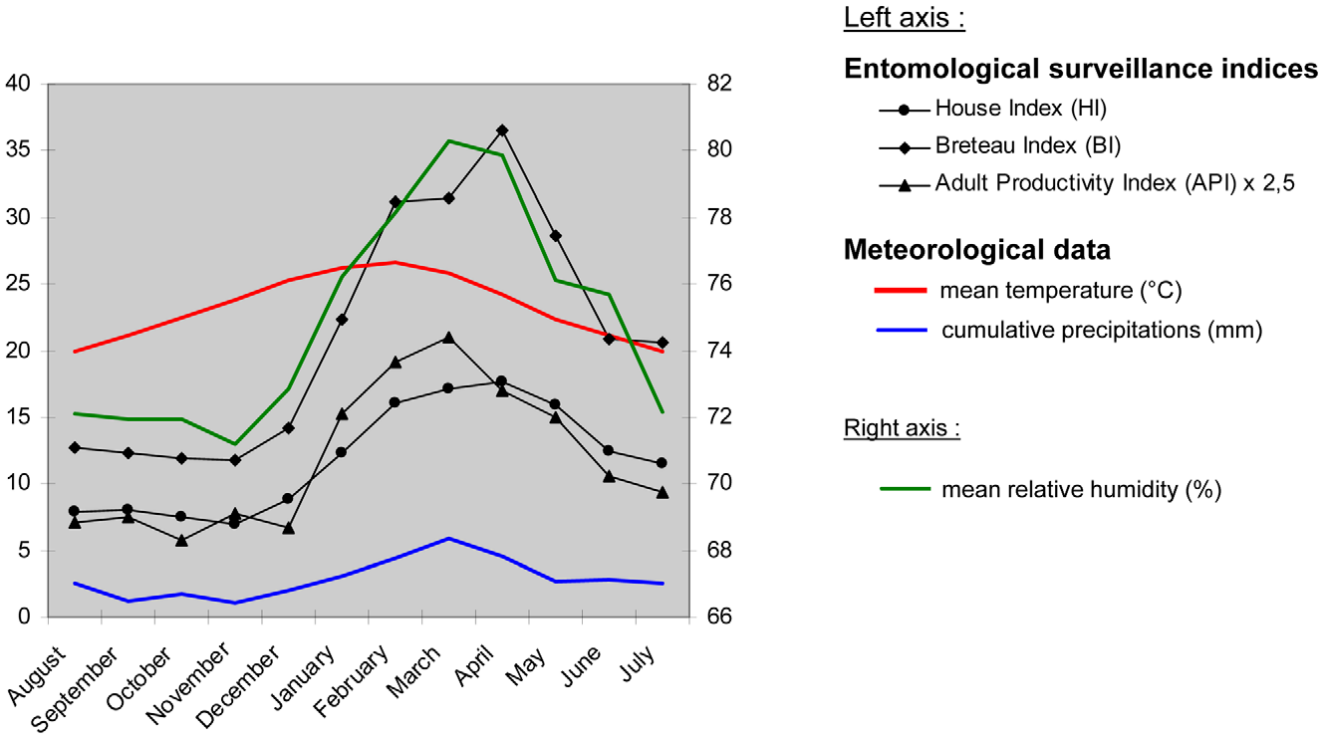
Seasonal evolution of monthly entomological surveillance indices and meteorological data in Noumea (August 2000–July 2009). HI, BI and API evolution display a strong seasonal cycle, with highest values between January and July. Entomological surveillance indices were significantly correlated with meteorological data at the seasonal scale. The peak of mean Temp preceded the peak of Precip, mean RH and API with a lag of one month, and the peak of HI and BI with a lag of two months.

Monthly means of HI, BI and API revealed a strong seasonal pattern with highest values between January and July (Figure 3).

#### Meteorological data

Over the 1971–2010 period, time series of annual means of daily mean Temp, Precip, and mean RH were characterized by a strong inter-annual variability. A number of ENSO events were observed including the strongest El Niño events of the century (i.e. 1982–1983 and 1997–1998). A global upward linear trend of annual mean Temp (mean increase of 0.75°C over the studied period, Figure 1) was observed in contrast with the Precip and mean RH time series that did not display any trend.

Rainfall is highly seasonal in New Caledonia. There are two main seasons: a warm and wet season (November–April), and a cooler and drier season (May–October). From November to April, max Temp in Noumea commonly reaches 30°C (on average during 42 days) and 6-month cumulative Precip 630 mm, whereas from May to October, max Temp rarely reaches 30°C (on average during only 2 days) and 6-month cumulative Precip are around 430 mm. The peak of mean Temp (February) precedes the peak of Precip and mean RH (March) with a lag of one month.

### Bivariate analysis

During the 1971–2010 period, a significant correlation was found between dengue incidence rates and mean annual mean Temp in Noumea (Spearman's coefficient *rho* = 0.426, *p*-value = 0.007, Figure 1) but there was no significant correlation with annual mean RH and Precip. Similar results were obtained with conserved trends and detrended data. Anomalies of annual means of mean Temp, Precip and mean RH were significantly correlated with ENSO, as measured by Niño 3.4 (*rho* = −0.365, *p*-value = 0.029; *rho* = −0.481, *p*-value = 0.003; *rho* −0.486, *p*-value = 0.003, respectively). During El Niño (positive value of Niño 3.4), the weather was cooler and drier. During La Niña (negative value of Niño 3.4), the weather was warmer and wetter. However, no direct correlation was found between ENSO and dengue incidence rates at the inter-annual scale (*rho* = −0.106, *p*-value = 0.539). Dengue outbreaks occurred during either El Niño, La Niña or neutral phases of ENSO.

During the 2000–2009 period, dengue incidence rates, meteorological and entomological data were analyzed in Noumea at a monthly scale. A strong seasonal distribution of HI, BI and API was observed (Figure 3), and significant correlations were found between monthly entomological surveillance indices and climate variables (data not shown). Although the highest dengue incidence rates and the highest values of HI, BI and API were observed during the same period of the year (from January to July), no significant time-lagged correlation has been found between monthly entomological indices and dengue incidence rates reported in Noumea over the 2000–2009 period (supporting Figure S1). We did not find relevant entomological patterns during dengue outbreaks. Accordingly, entomological surveillance indices were not used for the modelling of dengue outbreak risk.

### Comparative analysis of epidemic and non epidemic years

Based on the tercile method, there were 13 epidemic years (dengue incidence rate in the upper tercile, i.e. >19.48 cases/10 000 inhabitants) and 13 non epidemic years (dengue incidence rate in the lower tercile, i.e. <4.13 cases/10 000 inhabitants). A detailed analysis was performed based on monthly and quarterly meteorological data measured from September (year *y*-1) to April (year *y*), i.e. four months before and after the outbreak onset.

**Figure 4:**
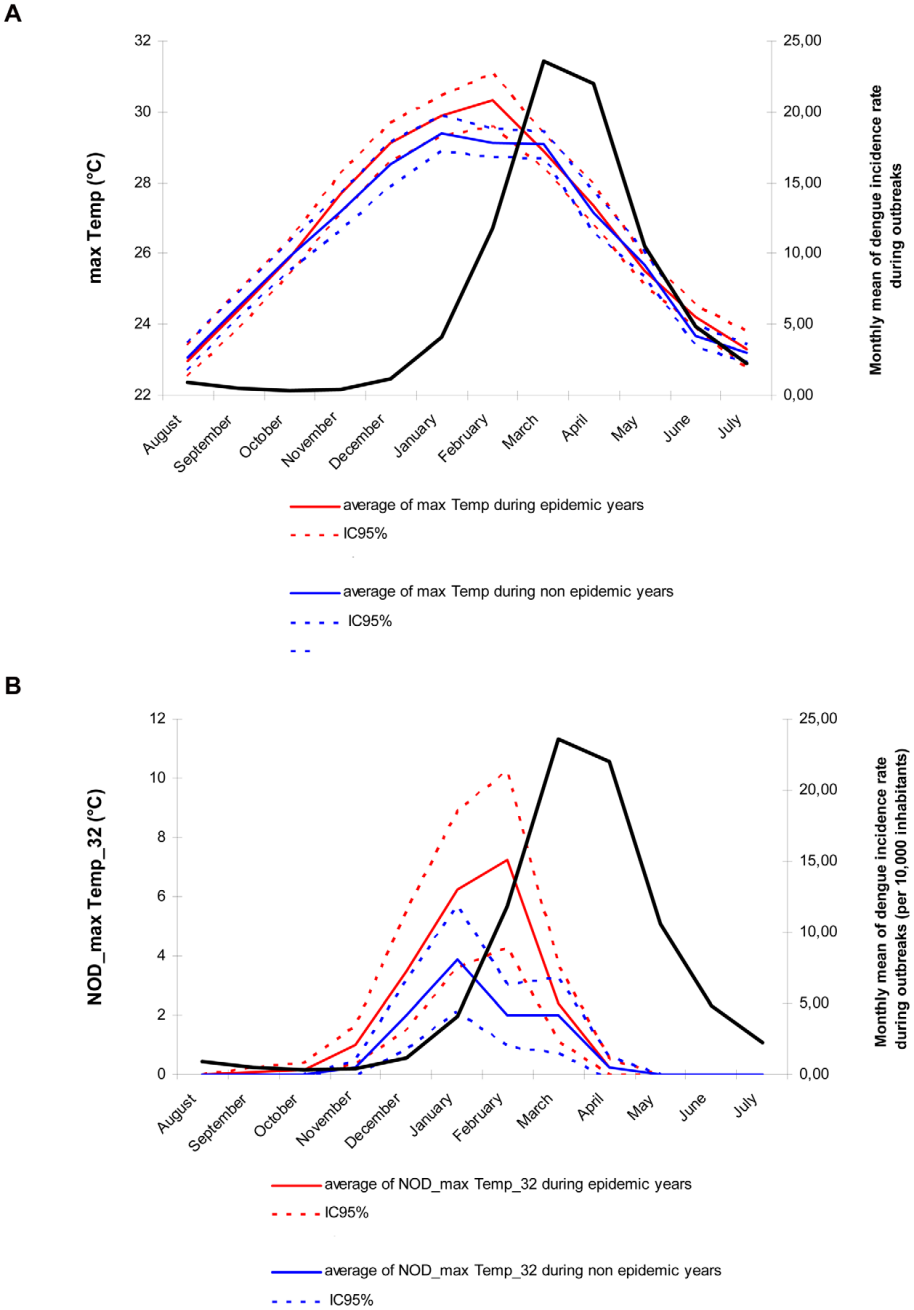
Relationship between maximal temperatures and dengue outbreaks in Noumea. Averages and 95% confidence intervals (IC95%) of max Temp (Figure 4a) and NOD_max Temp_32 (Figure 4b) calculated monthly during epidemic and non epidemic years were compared from August (year *y*-1) to July (year *y*). The peak of max Temp preceded the epidemic peak of dengue with a lag of 1–2 months. The number of days with max Temp exceeding 32°C during the first quarter of the year was significantly higher during epidemic years than during non epidemic years, especially in February (NOD_max Temp_32_February = 7.25 versus 2 days, respectively).

Temperatures (min Temp, mean Temp and max Temp) were higher during epidemic years than during non epidemic years. The peak of max Temp, observed usually in February, preceded the epidemic peak of dengue with a lag of 1–2 months (Figure 4a). Analysis of daily data allowed identifying important temperature thresholds. It revealed that the number of days with max Temp exceeding 32°C, mean Temp exceeding 27°C, and min Temp exceeding 22°C were significantly higher during epidemic years than during non epidemic years. The most important and significant differences were observed during the first quarter of the year, principally in February for max Temp (*p-value*<0.01 using a *t*-test, Figure 4b).

By contrast, the relationships between Precip, mean RH and dengue dynamics were not clear, as shown in supporting Figure S2. Highest Precip and mean RH were observed in February–March–April during the epidemic phase of dengue. Using a *t*-test, Precip and mean RH were significantly lower in February during epidemic years than during non epidemic years (*p*-value<0.01 and  = 0.04, respectively). Inversely, the ETP was significantly higher in February (*p*-value = 0.02). WF, HB, ENSO indices and entomological surveillance indices were not significantly different between epidemic and non epidemic years.

Meteorological variables showing strongest correlations with the epidemic years series, as defined in the Methods section, are presented for each family of variables in Table 1. Significant correlations were identified with several local meteorological variables (particularly Temp, Precip, RH, and ETP) but not with ENSO indices. No or poor correlation was found with WF and HB. In accordance with Figure 4 and supporting Figure S2, Temp were positively correlated with dengue outbreaks in Noumea, whereas Precip and RH measured in February were negatively correlated with dengue outbreaks. A positive correlation was found between the ETP measured in February and the occurrence of dengue outbreaks.

**Table 1:** Correlations between meteorological variables and dengue outbreaks in Noumea.

	Spearman's rank correlation test	
	*rho* coefficient	*p*-value
**Temperature (°C)**		
NOD_min Temp_22_JFM	0.58	<0.01
NOD_mean Temp_27_NDJ	0.59	<0.01
NOD_max Temp_32_JFM	0.51	<0.01
**Relative humidity (%)**		
NOD_min RH_70_February	−0.47	0.01
NOD_max RH_95_February	−0.47	0.01
NOD_max RH_80_SON	0.47	0.02
**Precipitations (mm)**		
Precip_February	−0.57	<0.01
NOD_Precip_0.1_December	−0.43	0.03
NOD_Precip_10_February	−0.41	0.04
**Potential evapotranspiration (mm)**		
ETP_February	0.44	0.02
NOD_ETP_4_February	0.50	0.01
NOD_ETP_6_FMA	0.47	0.01
**Hydric balance sheet (mm)**		
NOD_HB_10_February	−0.45	0.02
NOD_HB_0.5_FMA	−0.37	0.06
NOD_HB_5_ DJF	−0.32	0.11
**Wind Force (m/s)**		
NOD_WF_3_NDJ	0.41	0.04
NOD_WF_7_SON	0.37	0.07
WF_September	0.23	0.26
**ENSO**		
NINO.3.4_November	0.20	0.33
NINO.3.4_ASO	0.19	0.34
NINO.4_November	0.18	0.37

Monthly and quarterly meteorological data measured from September (year *y*-1) to April (year *y*), i.e. four months before or after the outbreak onset, were analyzed from 1971 to 2010 in Noumea. For each family of meteorological variables, the three variables most correlated with the occurrence of dengue outbreaks are presented, *p*-value<0.05 indicating statistical significance.

Monthly and quarterly parameters were named “parameter_month”, and “parameter_first letter of each month of the quarter”, respectively. Number of days with a parameter over a threshold *x* were named NOD_parameter_threshold *x*.

### Multivariate modelling of dengue outbreak risk

**Figure 5:**
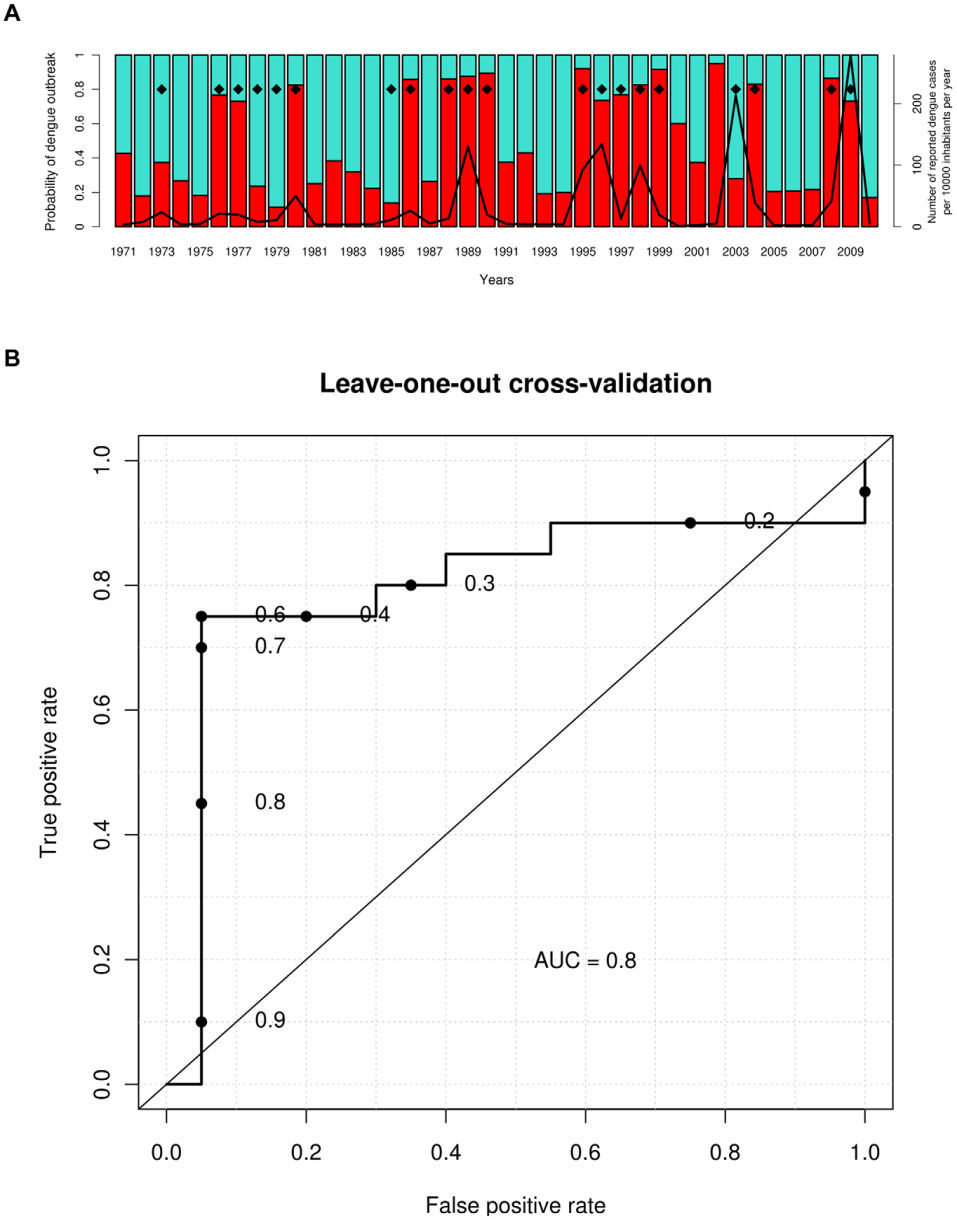
SVM explicative model of dengue outbreaks in Noumea (leave-one-out cross validation). The model estimates the probability of dengue outbreak occurrence (red bars) each year according to the number of days with maximal temperature exceeding 32°C during the first quarter of the year (NOD_max Temp_32_JFM), and the number of days with maximal relative humidity exceeding 95% during January (NOD_max RH_95_January). Results obtained in leave-one-out cross validation are presented in Figure 5a. The black line indicates the annual dengue incidence rate, and black diamonds indicate epidemic years according to the median method. The ROC curve (Figure 5b) indicates the rates of true and false positives for different detection thresholds. For example, for a probability of dengue outbreak above 65% (0.65), 15 of 20 epidemic years are predicted correctly (true positive rate = 75%) with only one false alarm (false positive rate = 5%). The sensitivity of the model for this threshold is 75% (15 epidemic years predicted correctly/20 epidemic years), the specificity 95% (19 non epidemic years predicted correctly/20 non epidemic years), the positive predictive value 94% (15 epidemic years predicted correctly/16 epidemic years predicted by the model), and the negative predictive value 79% (19 non epidemic years predicted correctly/24 non epidemic years predicted by the model).

**Figure 6:**
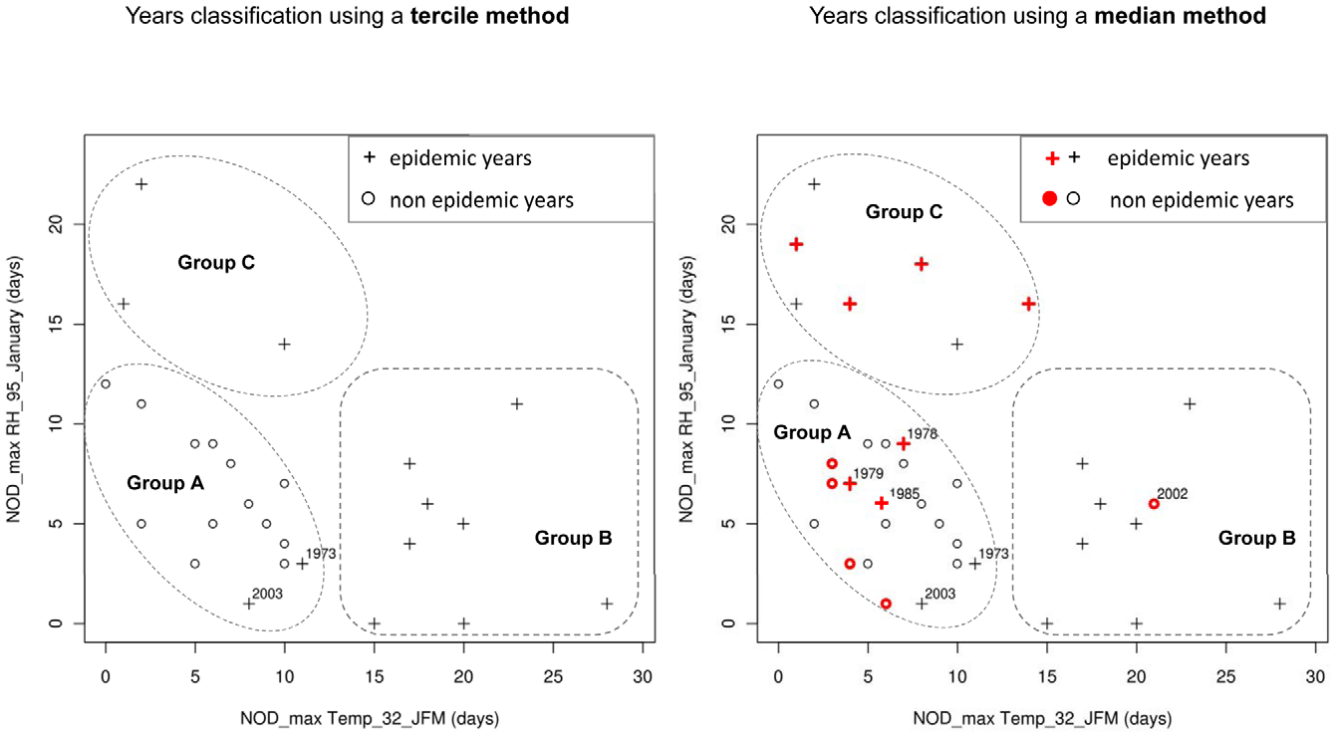
Scatter plots of epidemic and non epidemic years with regards to NOD_max Temp_32_JFM and NOD_max RH_95_January. Each year, the number of days with maximal temperature exceeding 32°C during January–February–March (NOD_max Temp_32_JFM) and the number of days with maximal relative humidity exceeding 95% during January (NOD_max RH_95_January) were calculated. Two methods denoted “tercile method” and “median method” were used to separate the years on the basis of annual dengue incidence rates in Noumea (see Methods). On the left panel, epidemic years (dengue incidence rate in the upper tercile, i.e. >19.48 cases/10,000 inhabitants/year) and non epidemic years (dengue incidence rate in the lower tercile, i.e. <4.13 cases/10,000 inhabitants/year) are presented. The distribution of crosses (epidemic years) and circles (non epidemic years) permits the identification of three groups (A, B, C). All non epidemic years belonged to group A whereas all epidemic years, except 1973 and 2003, belonged to either group B or group C suggesting that dengue outbreaks can occur in distinct climatic conditions. On the right panel, epidemic years (dengue incidence rate greater than the median, i.e. 7.65 cases/10,000 inhabitants/year) and non epidemic years (dengue incidence rate lower than the median) are presented with the advantage of a whole set of data being usable for modelling. Years that were not considered with the tercile method (dengue incidence rate in the middle tercile) are coloured in red. Further epidemic (red crosses) and non epidemic years (red circles) are considered with the median method, and similar groups (A, B, C) were identified. With the median method, three epidemic years (1978, 1979 and 1985) and one non epidemic year (2002) were incorrectly classified. These four years were characterized by annual dengue incidence rates closed to the median.

First, in order to produce an explicative model of dengue outbreak, we selected meteorological variables observed within the period of dengue outbreak onset, i.e. from January to April (Figure 2). The best SVM model based on the minimum AIC_c_ (−79.21) was obtained using two meteorological variables, i.e. the number of days with maximal temperature exceeding 32°C during the first quarter of the year (NOD_max Temp_32_JFM), and the number of days with maximal relative humidity exceeding 95% during January (NOD_max RH_95_January). The addition of a third meteorological variable did not improve the performance of the model. Results obtained in leave-one-out cross validation (Figure 5) were close to those obtained with the complete dataset (Figure S3) and were characterized by a high ROC-AUC value reaching 0.80 and 0.85, respectively. As indicated by the ROC curves, most of epidemic years were predicted correctly with high probability and few false alarms. Importantly, with bivariate analysis, NOD_max Temp_32_JFM was positively correlated with the occurrence of dengue outbreak (*rho* = 0.57, *p*-value = 0.002) whereas NOD_max RH_95_January did not appear to be a discriminatory meteorological variable (*rho* = −0.11, *p*-value = 0.58). With multivariate analysis, these two variables were highly informative and discriminatory. Scatter plots of epidemic and non epidemic years as a function of these two variables allowed the identification of three distinct groups (Figure 6): group A including years characterized by low NOD_max Temp_32_JFM (<12 days) and low NOD_max RH_95_January (<12 days), group B including years characterized by high NOD_max Temp_32_JFM (>12 days) and low NOD_max RH_95_January, and group C including years characterized by low NOD_max Temp_32_JFM and high NOD_max RH_95_January (>12 days). According to the tercile method of years classification, all non epidemic years belonged to group A whereas all epidemic years, except 1973 and 2003, belonged to either group B or group C. Similar results were obtained using the median method ensuring the inclusion of all years, preferable for the development of SVM models. Only four years (1978, 1979, 1985, and 2002) belonging to the middle tercile (dengue incidence rate ranging from 4.13 to 19.48 cases/10 000 inhabitants/year) were incorrectly classified using the median method. In 2002, although favorable climatic conditions for dengue outbreak were observed, the incidence rate (5.24 dengue cases/10 000 inhabitants/year) was close to the median (7.65 dengue cases/10 000 inhabitants/year). In 1978, 1979 and 1985, the low values of NOD_max Temp_32_JFM and NOD_max RH_95_January were not favorable for dengue outbreak. However, incidence rates (7.74, 10.63, and 11.24 dengue cases/10 000 inhabitants/year, respectively) were close to the median. Two years (1973 and 2003) belonging to epidemic years using either a tercile or a median method of classification were characterized by low NOD_max RH_95_January and intermediate NOD_max Temp_32_JFM, as members of group A (non epidemic years). However, dengue outbreaks occurred with high incidence rates (23.64 and 213.58 dengue cases/10 000 inhabitants/year in 1973 and 2003, respectively). These mismatches indicate that i) the model fails for years that are difficult to classify as their dengue incidence rates were close to the median and in the middle tercile and, ii) NOD_max Temp_32_JFM and NOD_max RH_95_January alone cannot account for all dengue outbreaks (Figure 6). It is likely that other climate events and other factors influencing dengue dynamics contribute to the epidemic spread of dengue viruses during these peculiar years. We were thus able to build an efficient explicative model of dengue epidemics based on meteorological variables contemporaneous to the outbreak.

**Figure 7:**
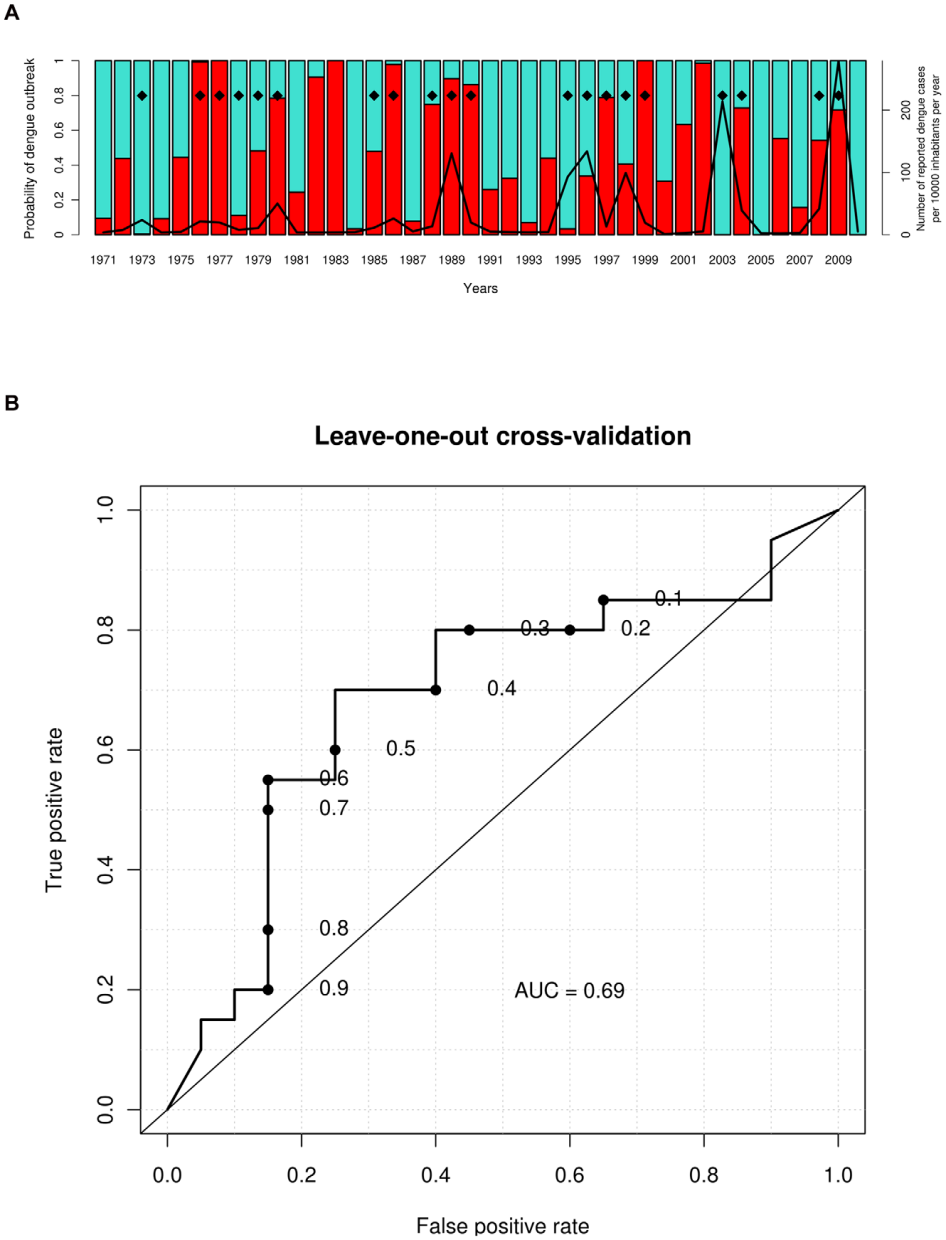
SVM predictive model of dengue outbreaks in Noumea (leave-one-out cross validation). The model estimates the probability of dengue outbreak occurrence (red bars) each year *y* according to the quarterly mean of maximal relative humidity during October–November–December (max RH_OND), and the monthly mean of maximal temperature in December (max Temp_December) year *y*-1. Results obtained in leave-one-out cross validation are presented in Figure 7a. The black line indicates the annual dengue incidence rate, and black diamonds indicate epidemic years according to the median method. The ROC curve (Figure 7b) indicates the rates of true and false positives for different detection thresholds. For example, for a probability of dengue outbreak above 65% (0.65), 11 of 20 epidemic years were predicted correctly (true positive rate = 55%) with three false alarms (false positive rate = 15%). The sensitivity of this model for this threshold is 55% (11 epidemic years predicted correctly/10 epidemic years), the specificity 85% (17 non epidemic years predicted correctly/20 non epidemic years), the positive predictive value 79% (11 epidemic years predicted correctly/14 epidemic years predicted by the model), and the negative predictive value 65% (17 non epidemic years predicted correctly/26 non epidemic years predicted by the model).

Another challenge was to construct a predictive model for dengue epidemics using variables available prior to the outbreak onset, i.e. from September (year *y*-1) to December (year *y*-1). Accurate predictive skill (AICc = −66.64) was achieved with the SVM model built from the value of the two following variables: the quarterly mean of maximal relative humidity during October–November–December (max RH_OND), and the monthly mean of maximal temperature in December (max Temp_December) of the year *y*-1 with a ROC-AUC value of 0.83 (supporting Figure S4). Probabilities obtained in leave-one-out cross validation (Figure 7) and the corresponding ROC-AUC value reaching 0.69 illustrate the robustness of this predictive model. Importantly, max RH_OND and max Temp_December were not significantly correlated with the risk of dengue outbreak with bivariate analysis (*rho* = 0.24, *p*-value = 0.14; and *rho* = 0.25, *p*-value = 0.14, respectively).

**Figure 8:**
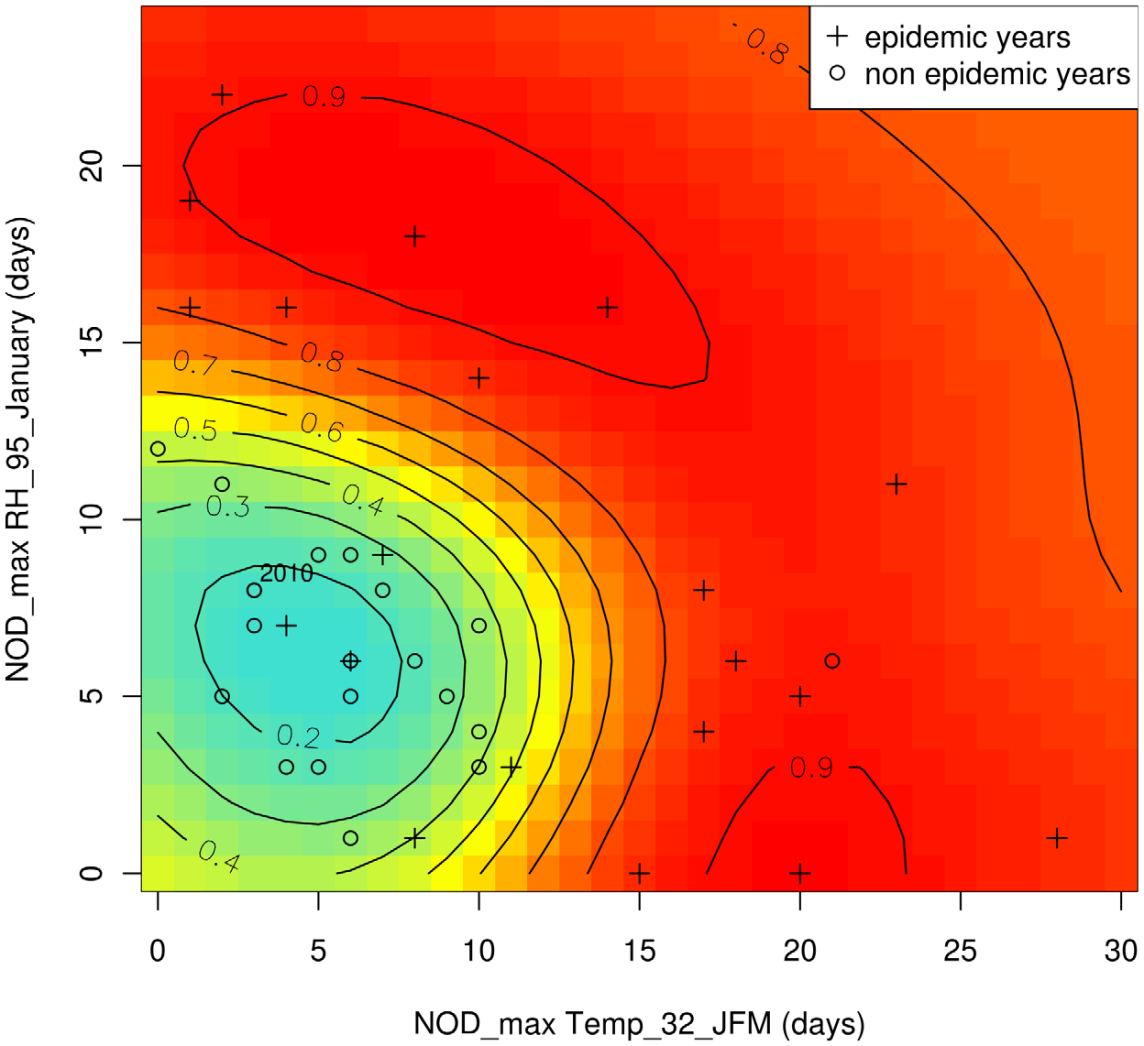
SVM explicative model probability contours superimposed with NOD_max Temp_32_JFM and NOD_max RH_95_January during epidemic/non epidemic years. Line-curves indicate the estimated probability of dengue outbreak occurrence given by the model. Blue colour indicates low risk, yellow colour indicates intermediate risk, and red colour indicates high risk of dengue outbreak. Meteorological parameters used to build the SVM models are shown for epidemic years (crosses) and non epidemic years (circles). The number of days with maximal temperature exceeding 32°C during January–February–March (NOD_max Temp_32_JFM) and the number of days with maximal relative humidity >95% during January (NOD_max RH_95_January) of the year *y* were used to build the SVM explicative model.

**Figure 9:**
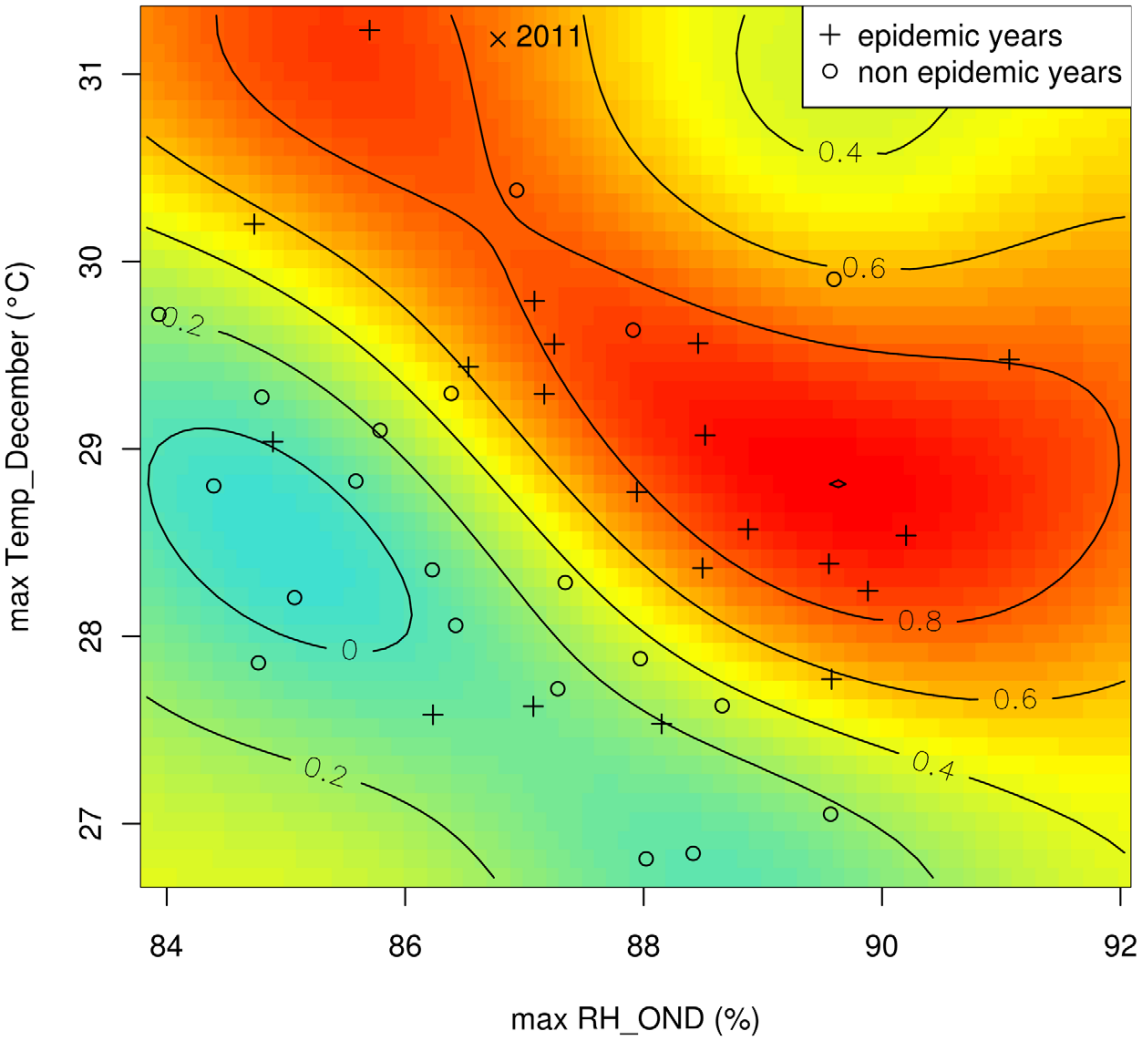
SVM predictive model probability contours superimposed with max RH_OND and max Temp_December during epidemic/non epidemic years. Similarly to the SVM explicative model (Figure 8), the quarterly mean of maximal relative humidity during October–November–December (max RH_OND), and the monthly mean maximal temperature in December (max Temp_December) of the year *y*-1 were used to build the SVM predictive model.

Scatter plots of epidemic years and non epidemic years built from the combination of meteorological variables used for the SVM explicative model (Figure 8) and for the SVM predictive model development (Figure 9) show that dengue outbreaks occurred in distinct climatic conditions in Noumea. With the SVM predictive model, as noted with the SVM explicative model, epidemic years belonged to two different groups of data according to the value of max RH_OND and max Temp_December (see the two red kernels corresponding to high risk of dengue outbreak in Figure 9). Dengue outbreaks occurred following either years characterized by high max Temp_December and relatively low max RH_OND, or years characterized by high max RH_OND_December, and max Temp_December. To note, the high value of max Temp_December (31.2°C) and the relatively low value of max RH_OND (86.8%) measured in 2010 indicate a high risk (74%) of dengue outbreak for 2011.

A comparison of the results obtained with the explicative model and the predictive model was performed together with a detailed analysis of the relationships between meteorological variables used to build the explicative model (NOD_max Temp_32_JFM and NOD_max RH_95_January) and those used to build the predictive model (max RH_OND and max Temp_December). As shown in Figure S5, strong relationships exist between the values of max Temp and max RH measured at the end of the year *y*-1, and those measured at the beginning of the year *y*. Low max RH_OND and max Temp_December (year *y*-1) were predictive of low NOD_max Temp_32_JFM and NOD_max RH_95_January (years *y*, group A). High max RH_OND and max Temp_December (year *y*-1) were predictive of either high NOD_max Temp_32_JFM and low NOD_max RH_95_January (years *y*, group B), or low NOD_max Temp_32_JFM and high NOD_max RH_95_January (years *y*, group C). Results obtained with the predictive model were highly consistent with those obtained with the explicative model with similar probabilities of dengue outbreak risk obtained for 30 of the 40 studied years. Failures of the predictive model can be explained by a lack of correlation between these meteorological variables on a few occasions (e.g. 1982, 1983, 1995). For example, although the predictive model estimated a risk of dengue outbreak close to 5% in 1995, the explicative model estimated a risk over 90%, and a major outbreak occurred. The value of max RH_OND and max Temp_December measured in 1994 (87% and 27.6°C, respectively) were relatively low and therefore not predictive of outbreak risk. However, climatic conditions were favorable for a dengue outbreak occurrence (NOD_max Temp_32_JFM = 20 days, NOD_max RH_95_January = 0 day, group B). This suggests that other climate variables or meteorological processes may impact on the local value of NOD_max Temp_32_JFM and NOD_max RH_95_January.

## Discussion

The influence of climate on dengue dynamics in Noumea, the capital of New Caledonia, over the 1971–2010 period has been analyzed at different time scales using high quality and high resolution meteorological observation data, along with epidemiological and entomological surveillance data. During epidemic years, dengue outbreaks peaked around March–April at the end of summer season. The epidemic peak lagged the warmest temperature by 1–2 months and was in phase with maximum precipitations and maximum relative humidity. The seasonal evolution of entomological indices (e.g, Breteau, House and Adult productivity indices) matched the seasonality of dengue outbreaks.

No relationship was found between the inter-annual variations of dengue incidence rates and those of the entomological data. On the other hand, a number of meteorological indices developed from summertime temperature, precipitation or relative humidity showed a significant correlation with dengue occurrence.

New explicative and operational predictive models of dengue outbreak were developed. We used a multivariate SVM model to identify the best set of meteorological variables explaining dengue epidemics. We found that a non linear combination of two meteorological variables strongly outperforms a model based on a single variable or a linear approach, as commonly employed in the literature. We found the best explicative variables to be the number of days with max Temp exceeding 32°C during January–February–March (NOD_max Temp_32_JFM) and the number of days with max RH exceeding 95% during January (NOD_max RH_95_January). When the model gives a probability of dengue outbreak above 65%, these two variables explain 94% of the epidemic years and 79% of the non epidemic years (Figure 5). Most dengue outbreaks occurred within two kinds of distinct climatic conditions: high NOD_max Temp_32_JFM and low NOD_max RH_95_January, or low NOD_max Temp_32_JFM and high NOD_max RH_95_January. We were also able to build another SVM model based on two variables to predict dengue outbreaks in advance: the maximal temperature in December (max Temp_December) and maximal relative humidity during October–November–December (max RH_OND) of the year prior to the epidemics. For a probability of dengue outbreak above 65%, this model can predict 79% of the epidemic years and 65% of the non epidemic years (Figure 7).

### Influence of local meteorological conditions on dengue dynamics

Overall, the high performance of the climate-based models of dengue outbreak risk developed in our study suggest that dengue dynamics were essentially driven by climate during this 1971–2010 period in Noumea. The explicative model provides important and new information. We have shown that maximal values of temperature and relative humidity were determinant in dengue outbreaks occurrence and precise thresholds of their value were identified. Importantly, we found that the most relevant meteorological variables explaining dengue outbreaks were built using the number of days for which the variable was greater than a threshold value introducing the importance of the persistence of suitable climatic conditions. Our findings are compatible with the mosquito biology and viral transmission cycle.

The length of *Aedes* gonotrophic cycle is shorter at temperatures above 32°C and feeding frequency is more than twofold at 32°C as compared to 24°C; pupae development period reduced from four days at 22°C to less than one day at 32–34°C [16]–[17], [47]. Additionally, the experimental infection of *A. aegypti* with DENV-2 viruses showed that the extrinsic incubation period shortens from 12 days at 30°C to seven days at 32–35°C leading to an increasing risk of viral transmission from an infected mosquito to a susceptible host [15]. The influence of temperature on the rate of virus replication inside mosquitoes was also evidenced in the study of Watts *et al*. Temperatures may also influence the vector size and its biting rate [19], [21]. Consequently, it is likely that the increased level of viral transmission characterizing dengue outbreaks in Noumea at temperatures exceeding 32°C may be a consequence of shortening of the *A. aegypti* gonotrophic cycle and extrinsic incubation period, and of increased vector feeding frequency.

Mortality rate of larvae, pupae and adult mosquitoes as a function of temperature between 10 and 40°C can be represented by a wide-base ‘U’ graphical shape with lower mortality rate at temperature ranging from 15 to 30°C [16]–[20], [22]. Hence, *A. aegypti* mortality rate may be relatively constant at temperatures observed usually in Noumea, and the increasing mortality rate expected above 32°C is not likely to be an important limiting parameter in the spread of dengue viruses in this specific ecosystem.

Larval breeding places are mostly outdoors in Noumea and mosquito abundance increases during the rainy and humid season. Moreover, relative humidity may be determinant in *A. aegypti* egg development and adult population size that may itself be correlated with vectorial capacity [48]. High humidity shortens incubation and blood-feeding intervals; it favours adult mosquito longevity [20] and thus dengue transmission. This may explain why a sustained high RH during January is associated with a higher risk of dengue outbreak in Noumea.

### Influence of remote climate conditions on dengue dynamics

On a broader scale, a growing number of studies have shown that ENSO may be associated with changes in the risk of mosquito borne diseases such as dengue [23]–[24]. By contrast, Hales *et al.* [31] further analyzed the relationships between the annual number of dengue cases in New Caledonia, ENSO, temperature and rainfall using global atmospheric reanalyses climate based data, and they did not find any significant correlation between SOI and dengue (Pearson's coefficient = 0.20). In accordance with this study, and with the advantage of observational and long term data, we found significant inter-annual correlations between ENSO and our local climate but not between ENSO and dengue (Table 1). Moreover, the selection process of multivariate models did not select any ENSO index neither in explicative mode nor in predictive mode. These findings suggest that, in New Caledonia, large-scale climate indices such as ENSO cannot account for the complexity of the local meteorological inter-annual situations. However, at a larger scale, Hales *et al.* showed that the number of dengue outbreaks in the South Pacific islands (aggregated data, 1970–1995) were positively correlated with the SOI [30], suggesting that La Niña may favour dengue outbreaks in this region of the world. The impact of ENSO on local weather in the South Pacific may strongly vary from one place to another. New Caledonia, located around 20° south latitude in the western Pacific is relatively far from the main centre of action of ENSO located in the equatorial central/eastern equatorial Pacific and its local weather is thus not only influenced by ENSO, but also by other climate modes such as the Madden-Julian Oscillation which strongly influences local meteorological parameters at intra-seasonal (30 to 90 days) time scales [49]. In contrast, ENSO influence may be stronger in islands located closer to the equator, the relationship between ENSO and dengue epidemics being therefore more straightforward [29].

Our long-term study also suggests an increasing risk of dengue outbreaks in New Caledonia in the context of global warming (Figure 1). Even though a global upward trend of dengue incidence rates was noted along the 1971–2010 period, and as surveillance methods and laboratory tests have evolved, it is difficult to know if the amplitude of dengue outbreaks is significantly growing.

### Dengue dynamics driven by multiple factors

Even though climate influenced the disease epidemiology in Noumea during this forty-year period, the reasons of dengue emergence in New Caledonia are multiple, including population growth (119,710 inhabitants in 1973 to 245,580 in 2009), accelerated urbanization particularly around Noumea, tourism development and increasing international and inter-islands traffic [50]. The emergence of dengue fever in other parts of the world, particularly South East Asia where dengue is endemic with a co-circulation of the four serotypes, represents an increasing source of virus introduction into New Caledonia. Indeed, multiple and repeated introductions of dengue viruses have been detected from several countries in Asia [34]. Moreover, the geographical distribution of *A. aegypti* has expanded during recent decades in New Caledonia (Paupy and Guillaumot, unpublished data).

Well known factors may have contributed to the epidemic dynamics such as the size of susceptible human hosts and vectors populations. In the absence of seroprevalence data, and due to the lack of long term entomological data, these variables were not included in the input dataset of the models. Nevertheless, as dengue is known to confer a prolonged serotype-specific immunity in the long term, herd immunity represents an important factor in understanding dengue dynamics [51]–[54]. In New Caledonia, successive waves of dengue outbreaks involving the same serotype were reported in 1980 and 1986 (DENV-4), 1989 and 1995 (DENV-3), 2003 and 2008 (DENV-1). This constant interval time between two epidemics involving the same serotype has already been observed in other South Pacific Islands [55]–[57]. Recently, a large molecular characterization of DENV-1 viruses collected regularly in French Polynesia between the 2001 and 2006 outbreaks revealed that the virus responsible for the severe 2001 outbreak was introduced from South-East Asia, and evolved under an endemic mode until its re-emergence under an epidemic mode five years later [56]. These findings suggest that 5–6 years may be necessary for the renewal of the susceptible population in these islands. In New Caledonia, at four occasions, dengue outbreaks were detected between January and July during two successive years: in 1976–1977 (DENV-1), 1995–1996 (DENV-3), 2003–2004 (DENV-1), and 2008–2009 (DENV-1 and DENV-4). This suggests that environmental conditions may be not favorable for dengue transmission all through the epidemic year, particularly during the second semester of the year characterized by lower values of entomological indices. It is likely that dengue re-emerged the following year when climatic conditions were favorable for dengue transmission (as suggested by the results of our explicative model in 1977, 1996, 2004 and 2009) and the size of the mosquito-vector and susceptible human populations were still sufficient for a large spread of dengue viruses. In these four examples of recurrent outbreaks during two consecutive years, it is more likely that the end of the epidemic was driven by limiting climatic factors and intricate entomological factors rather than by the depletion of the susceptible population.

The relationship between *Aedes* density and the intensity of dengue transmission remains unclear [47], [58]–[60]. Although dengue viruses cannot circulate if mosquito vectors are not present, the vector density of adult female *A. aegypti* necessary for dengue viruses to become endemic or epidemic remains unknown. In Noumea, entomological indices (HI, BI and API) were not correlated with the incidence rate of dengue, they were sometimes lower during epidemic than during non epidemic periods and lowest values were measured during the largest outbreak in 2009. The fact that these usual entomological surveillance indices (particularly API) are good indicators of adult density in Noumea suggests that the mosquito density threshold under which dengue viruses cannot spread widely may be very low and has never been reached up to now. Moreover, mosquito populations are influenced by human behaviours and meteorological variables alone cannot account for their geographical distribution and abundance [14], [61]. At the domestic level, *A. aegypti* populations are also influenced by global trends in urbanization, socioeconomic conditions, and vector control efforts. For instance, the outbreak predicted in 2002 with a probability close to 90% did not occur. A possible explanation is that strong vector control policies (e.g. increased efforts to reduce mosquito breeding sites and undertake human population education, development of perifocal spraying of insecticides) were undertaken in New Caledonia at the time of large dengue outbreaks in the other Pacific French overseas territories (French Polynesia in 2001, Wallis and Futuna in 2002). A relaxation in vector control efforts at the end of 2002 may have allowed the resurgence of dengue in the East coast and the spread of the virus through the archipelago during the next year.

Overall, our results suggest that the local climate had a major effect on dengue dynamics in Noumea during the last forty years. It is likely that other factors, not included in the input dataset of the models, had a lower influence on dengue epidemic dynamics. The introduction of dengue viruses may have been relatively constant, and the number of human hosts susceptible to a given serotype and of mosquito-vectors may have been always sufficient for an epidemic to occur when suitable climate conditions were met. It is likely that the susceptibility of human populations influenced the serotype involved in the outbreak and the epidemic magnitude. The variability of the length of the gonotrophic cycle, the extrinsic incubation period, and the life span of infected mosquitoes under climate change rather than the overall vector density may play a major role on the epidemic dynamics of dengue at the seasonal scale.

### Epidemics forecasting model

Although the meteorological variables contemporaneous to the epidemic season provide crucial information on local dengue dynamics as discussed above, prediction models are needed to anticipate the risk before the dengue outbreak onset and to make the model useful for health authorities in New Caledonia. In this study, we were able to build such a predictive model relying on maximal temperature and relative humidity measured in Noumea at the end of the previous year.

Biological interpretations about statistical associations between specific climatic conditions and the yearly risk of dengue outbreak in Noumea can be made in the frame of the explicative model as it uses relevant climatic variables that occur within the period of outbreak onset. The meteorological variables selected in the frame of the predictive model are tightly connected with the explicative meteorological variables (Figure S5).

As Noumea concentrates the majority of inhabitants and of dengue cases, as this city has been affected by all dengue outbreaks that occurred in New Caledonia during the last 40 years, and as dengue epidemics usually begin in Noumea, our predictive model is useful to anticipate the risk of dengue outbreak in New Caledonia. However, climatic conditions in Noumea can not account for dengue epidemics in other localities in New Caledonia that would not involve Noumea, even if this situation has never been observed in 40 years.

Depending on the user's objectives, different detection thresholds corresponding to a probability of dengue outbreak can be used. In the case of dengue, it is likely that decision makers would prefer to choose a detection threshold with high true positive rate and low false positive rate, as obtained with a detection threshold of 65% (Figure 7b). The model initialized in December 2009 indicated no risk of dengue outbreak for 2010 that was in accordance with the current epidemiological situation. To note, a high risk of dengue outbreak is predicted for 2011 (74%, Figure 9). Up to now, only a few cases of dengue fever have been reported. Only one case imported from the Philippines was possible to type and belonged to the serotype 1. It is likely that a significant part of the human population is immunized against the serotypes 1 and 4 involved in the largest dengue outbreaks reported in New Caledonia in 2008 and 2009 but the introduction of a new serotype (DENV-2 or DENV-3) may lead to another epidemic. However, several important confusing factors may interfere with dengue dynamics this year such as the massive rainfalls brought by the tropical cyclone Vania in middle January 2011 with its unknown effects on vector populations, the introduction and worrying local diffusion of Chikungunya viruses transmitted by the same mosquito and the subsequent enhancement of vector control policies.

### Conclusions and perspectives

In conclusion, the epidemic dynamics of dengue fever were strongly influenced by climate variability in Noumea during the 1971–2010 period. Local thresholds of maximal temperature and relative humidity have been identified with precision allowing the development of explicative and predictive climate-based models of dengue outbreak risk. The health authorities of New Caledonia have now integrated these models into their new decision making process in order to improve their management of dengue, in combination with clinical, laboratory (e.g. serotype determination), and entomological surveillance data. This work provides an example of the practical utility of research projects in operational public health fields and reinforces the need for a multidisciplinary approach in the understanding and management of vector-borne diseases. Our results provide also new insights for future experimental studies. It seems important now to study the impact of maximal temperatures exceeding 32°C and maximal relative humidity exceeding 95%, and the influence of their duration (more or less than 12 days) on the length of the extrinsic incubation period, feeding frequency and longevity of *A. aegypti* from New Caledonia.

The epidemic dynamics of dengue are driven by complex interactions between human-hosts, mosquito-vectors and viruses. These interactions are influenced by environmental and climatic factors that may have more or less burden according to the geographical localisation, the local climatic conditions, the vector characteristics (e.g. *Aedes* species and strains), the size and movements of human populations and the epidemiology of dengue. Consequently, our results can not be applied to other ecosystems. However, the methodology of analysis used in this study could be extended to other localities highly threatened by the emergence of dengue in the South Pacific, like in other tropical and subtropical countries. As global atmospheric reanalyses climate based data exist, there is hope for the development of local predictive models of dengue outbreak in countries where no reliable weather data are available.

## Supporting Information

Figure S1 Evolution of House Index, Adult Productivity Index and dengue cases reported in Noumea (2000–2009). The monthly incidence rate of dengue cases (histograms) reported in Noumea from March 2000 to December 2009 was not significantly correlated (time-lag being equal to 0, 1, 2, or 3 months) with the value of HI (orange line) reflecting the abundance of larval resting places, and API (green line) reflecting the vector density. Although highest dengue incidence rates and highest values of entomological surveillance indices were observed during the same period of the year (from January to July), no relevant entomological patterns were identified during dengue outbreaks. A decreasing trend of entomological indices was observed that may reflect the impact of strengthened vector control policies. Sometimes, higher indices were measured during non epidemic than during epidemic years, and lowest indices were observed in 2009 whereas a major dengue outbreak occurred suggesting that the minimal vector density allowing the occurrence of dengue outbreaks may be very low.
(TIF)

Figure S2 Relationship between monthly cumulative precipitations, mean relative humidity and dengue outbreaks in Noumea. Averages and 95% confidence intervals (IC95%) of Precip (Figure S2a) and mean RH (Figure S2b) calculated monthly during epidemic and non epidemic years were compared from August (year *y*-1) to July (year *y*). Highest Precip and mean RH were observed during the epidemic phase of dengue.
(TIF)

Figure S3 SVM explicative model of dengue outbreaks in Noumea (complete dataset). The model estimates the probability of dengue outbreak occurrence (red bars) each year according to the number of days with maximal temperature exceeding 32°C during the first quarter of the year (NOD_max Temp_32_JFM), and the number of days with maximal relative humidity exceeding 95% during January (NOD_max RH_95_January). Results obtained with the complete dataset are presented in Figure S3a. The black line indicates the annual dengue incidence rate, and black diamonds indicate epidemic years according to the median method. The ROC curve (Figure S3b) indicates the rates of true and false positives for different detection thresholds.
(TIF)

Figure S4 SVM predictive model of dengue outbreaks in Noumea (complete dataset). The model estimates the probability of dengue outbreak occurrence (red bars) each year *y* according to the quarterly mean of maximal relative humidity during October–November–December (max RH_OND), and the monthly mean of maximal temperature in December (max Temp_December) of the year *y-*1. Results obtained with the complete dataset are presented in Figure S4a. The black line indicates the annual dengue incidence rate, and black diamonds indicate epidemic years according to the median method. The ROC curve (Figure S4b) indicates the rates of true and false positives for different detection thresholds.
(TIF)

Figure S5 Relationships between predictive climate variables (year *y*-1) and explicative climate variables (year *y*). Line-curves indicate the probability of dengue outbreak occurrence estimated by the SVM predictive model. Blue colour indicates low risk, yellow colour indicates intermediate risk, and red colour indicates high risk of dengue outbreak. The values of the quarterly mean of maximal relative humidity during October–November–December (max RH_OND), and the maximal temperature in December (max Temp_December) of the year *y*-1 used to build the SVM predictive model were calculated each year during the 1971–2010 period. The point coordinates were associated each year with the letter A, B, or C according to the value of the two climate variables used to build the SVM explicative model, i.e. the number of days with maximal temperature exceeding 32°C during January–February–March (NOD_max Temp_32_JFM) and the number of days with maximal relative humidity >95% during January (NOD_max RH_95_January). As in Figure 7, members of group A correspond to years *y* with a low NOD_max Temp_32_JFM and a low NOD_max RH_95_January. Members of group B correspond to years *y* with high NOD_max Temp_32_JFM and low NOD_max RH_95_January. Members of group C correspond to years *y* with high NOD_max Temp_32_JFM and high NOD_max RH_95_January. Most of members of the group A correspond to non epidemic years whereas most of members of the group B or C correspond to epidemic years. This figure illustrates the strong relationship existing between the predictive and the explicative climate variables used to build the models. Low max RH_OND and max Temp_December (year *y*-1) were predictive of low NOD_max Temp_32_JFM and NOD_max RH_95_January (years *y*, group A). High max RH_OND and max Temp_December (year *y*-1) were predictive of either high NOD_max Temp_32_JFM and low NOD_max RH_95_January (years *y*, group B), or low NOD_max Temp_32_JFM and high NOD_max RH_95_January (years *y*, group C).
(TIF)
