# Supplementary figures and images for: Climate-Based Models for Understanding and Forecasting Dengue Epidemics
Source: PLoS Negl Trop Dis. 2012 Feb 14;6(2):e1470. doi: 10.1371/journal.pntd.0001470 (PMC3279338; doi:10.1371/journal.pntd.0001470)

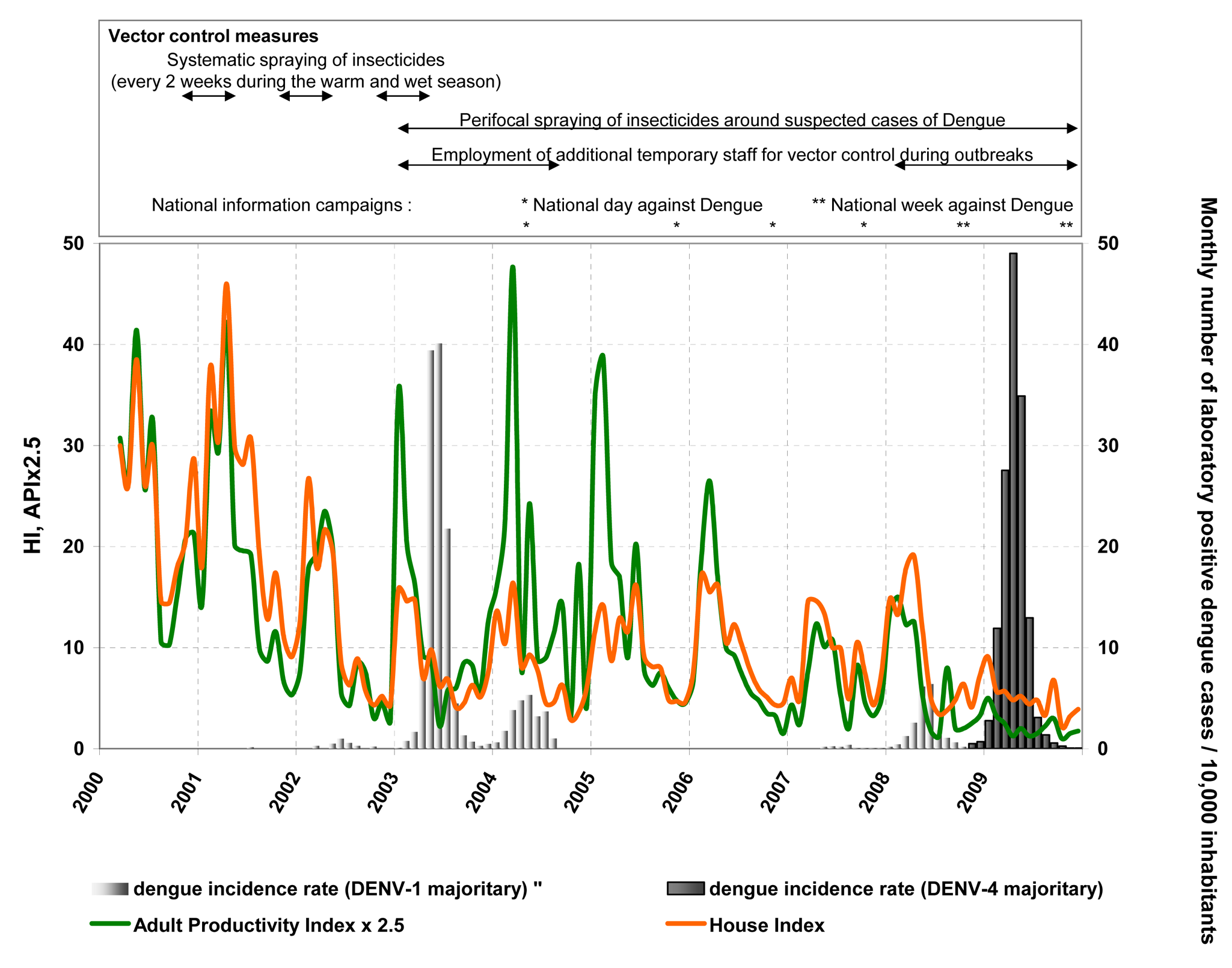

Supplement: Figure S1 — Evolution of House Index, Adult Productivity Index and dengue cases reported in Noumea (2000–2009). The monthly incidence rate of dengue cases (histograms) reported in Noumea from March 2000 to December 2009 was not significantly correlated (time-lag being equal to 0, 1, 2, or 3 months) with the value of HI (orange line) reflecting the abundance of larval resting places, and API (green line) reflecting the vector density. Although highest dengue incidence rates and highest values of entomological surveillance indices were observed during the same period of the year (from January to July), no relevant entomological patterns were identified during dengue outbreaks. A decreasing trend of entomological indices was observed that may reflect the impact of strengthened vector control policies. Sometimes, higher indices were measured during non epidemic than during epidemic years, and lowest indices were observed in 2009 whereas a major dengue outbreak occurred suggesting that the minimal vector density allowing the occurrence of dengue outbreaks may be very low. (TIF) [file pntd.0001470.s001.tif]

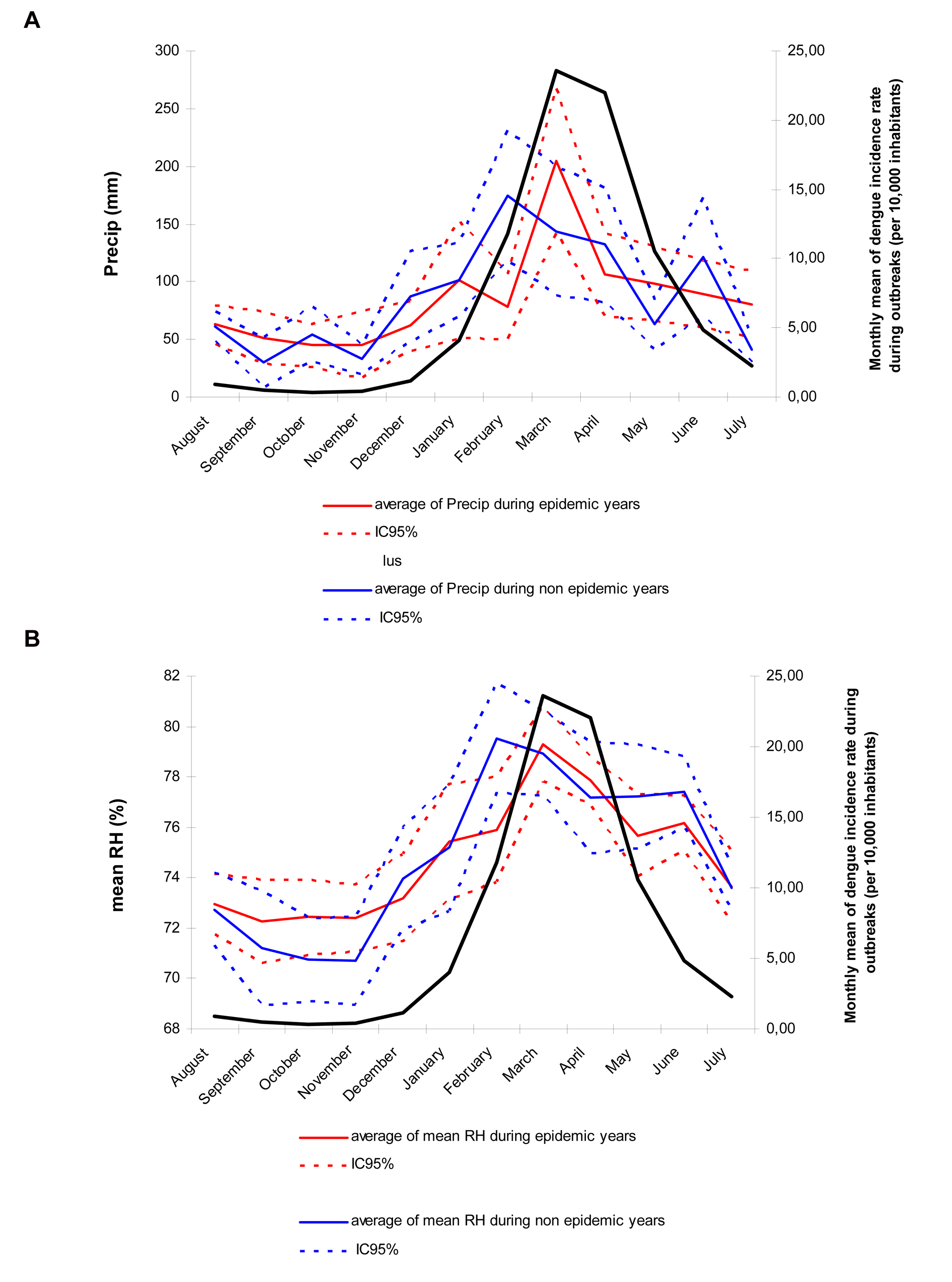

Supplement: Figure S2 — Relationship between monthly cumulative precipitations, mean relative humidity and dengue outbreaks in Noumea. Averages and 95% confidence intervals (IC95%) of Precip (Figure S2a) and mean RH (Figure S2b) calculated monthly during epidemic and non epidemic years were compared from August (year y-1) to July (year y). Highest Precip and mean RH were observed during the epidemic phase of dengue. (TIF) [file pntd.0001470.s002.tif]

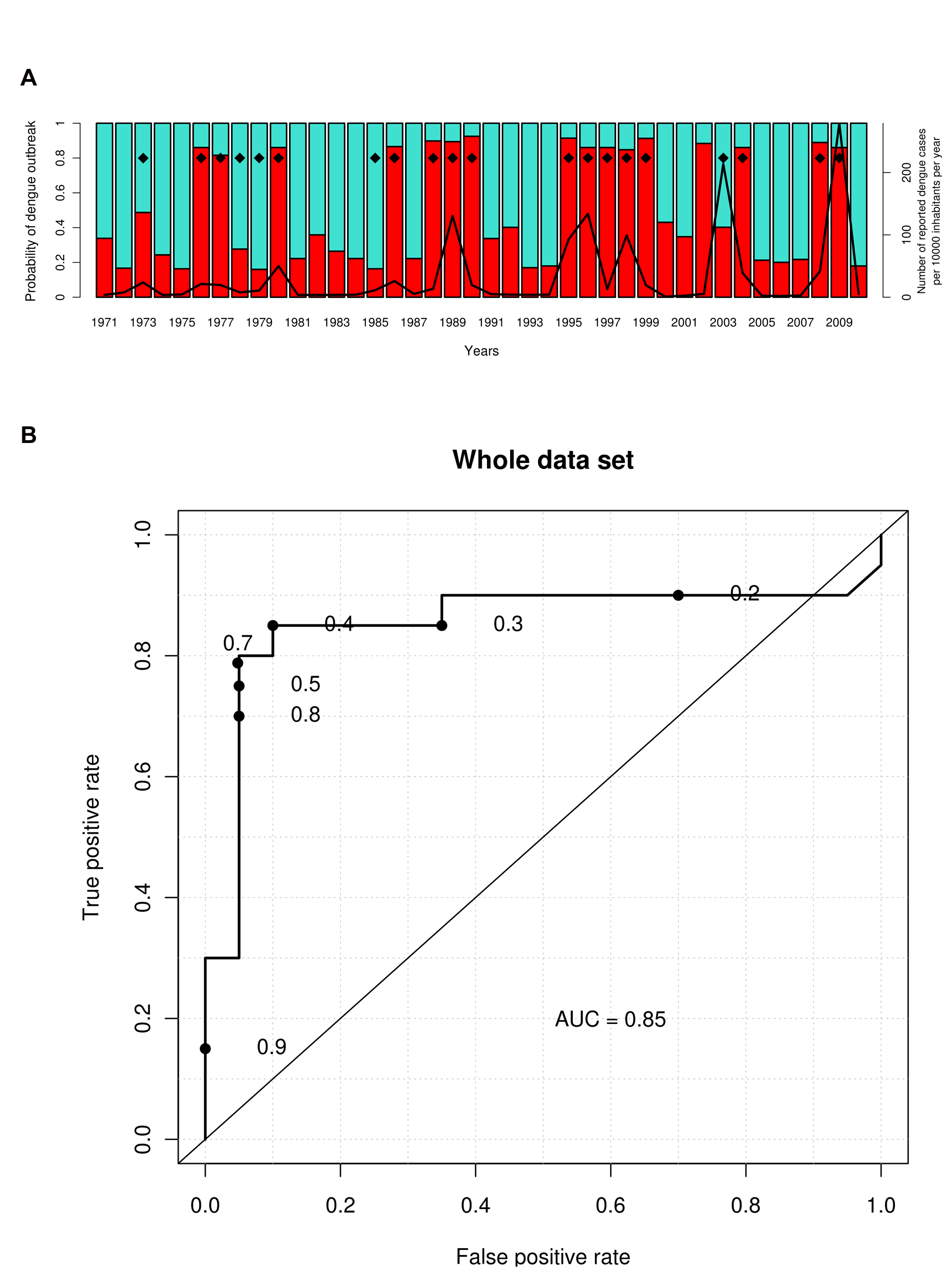

Supplement: Figure S3 — SVM explicative model of dengue outbreaks in Noumea (complete dataset). The model estimates the probability of dengue outbreak occurrence (red bars) each year according to the number of days with maximal temperature exceeding 32°C during the first quarter of the year (NOD_max Temp_32_JFM), and the number of days with maximal relative humidity exceeding 95% during January (NOD_max RH_95_January). Results obtained with the complete dataset are presented in Figure S3a. The black line indicates the annual dengue incidence rate, and black diamonds indicate epidemic years according to the median method. The ROC curve (Figure S3b) indicates the rates of true and false positives for different detection thresholds. (TIF) [file pntd.0001470.s003.tif]

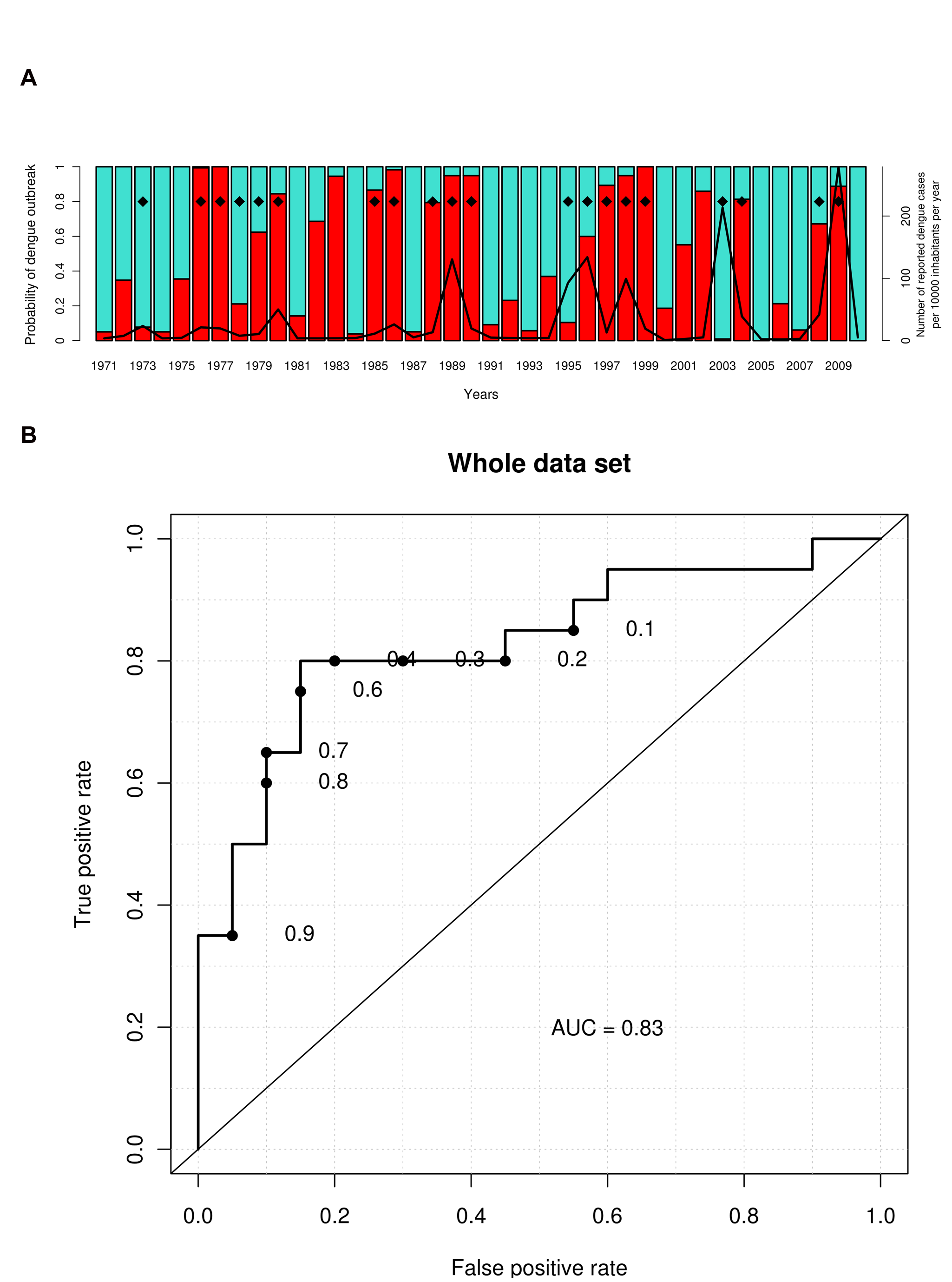

Supplement: Figure S4 — SVM predictive model of dengue outbreaks in Noumea (complete dataset). The model estimates the probability of dengue outbreak occurrence (red bars) each year y according to the quarterly mean of maximal relative humidity during October–November–December (max RH_OND), and the monthly mean of maximal temperature in December (max Temp_December) of the year y-1. Results obtained with the complete dataset are presented in Figure S4a. The black line indicates the annual dengue incidence rate, and black diamonds indicate epidemic years according to the median method. The ROC curve (Figure S4b) indicates the rates of true and false positives for different detection thresholds. (TIF) [file pntd.0001470.s004.tif]

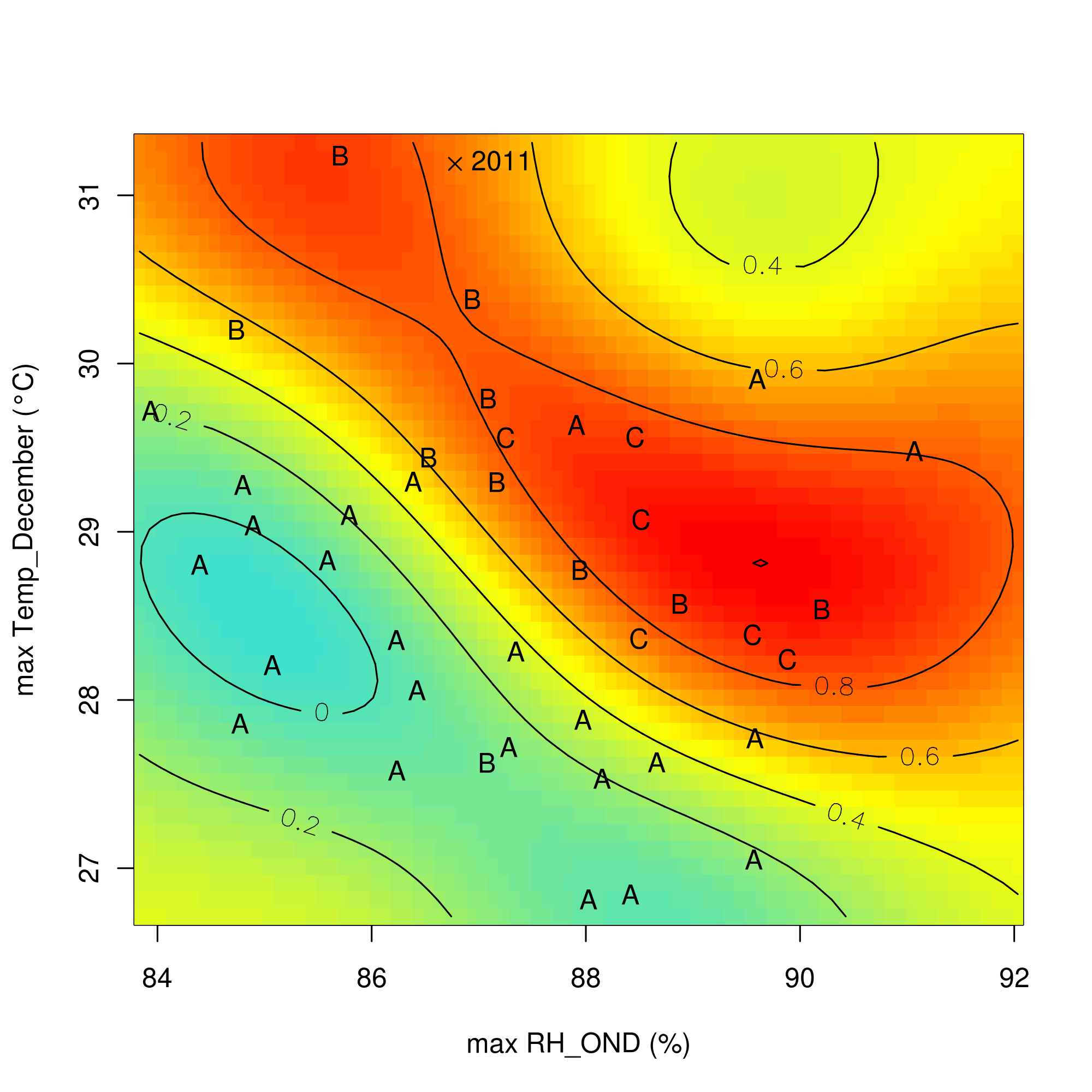

Supplement: Figure S5 — Relationships between predictive climate variables (year y-1) and explicative climate variables (year y). Line-curves indicate the probability of dengue outbreak occurrence estimated by the SVM predictive model. Blue colour indicates low risk, yellow colour indicates intermediate risk, and red colour indicates high risk of dengue outbreak. The values of the quarterly mean of maximal relative humidity during October–November–December (max RH_OND), and the maximal temperature in December (max Temp_December) of the year y-1 used to build the SVM predictive model were calculated each year during the 1971–2010 period. The point coordinates were associated each year with the letter A, B, or C according to the value of the two climate variables used to build the SVM explicative model, i.e. the number of days with maximal temperature exceeding 32°C during January–February–March (NOD_max Temp_32_JFM) and the number of days with maximal relative humidity >95% during January (NOD_max RH_95_January). As in Figure 7, members of group A correspond to years y with a low NOD_max Temp_32_JFM and a low NOD_max RH_95_January. Members of group B correspond to years y with high NOD_max Temp_32_JFM and low NOD_max RH_95_January. Members of group C correspond to years y with high NOD_max Temp_32_JFM and high NOD_max RH_95_January. Most of members of the group A correspond to non epidemic years whereas most of members of the group B or C correspond to epidemic years. This figure illustrates the strong relationship existing between the predictive and the explicative climate variables used to build the models. Low max RH_OND and max Temp_December (year y-1) were predictive of low NOD_max Temp_32_JFM and NOD_max RH_95_January (years y, group A). High max RH_OND and max Temp_December (year y-1) were predictive of either high NOD_max Temp_32_JFM and low NOD_max RH_95_January (years y, group B), or low NOD_max Temp_32_JFM and high NOD_max RH_95_January (years y, group C). (TIF) [file pntd.0001470.s005.tif]
